# Antioxidant-Rich *Woodfordia fruticosa* Leaf Extract Alleviates Depressive-Like Behaviors and Impede Hyperglycemia

**DOI:** 10.3390/plants10020287

**Published:** 2021-02-03

**Authors:** Mohammed Abu Tayab, Kazi Ashfak Ahmed Chowdhury, Md. Jabed, Syed Mohammed Tareq, A. T. M. Mostafa Kamal, Mohammad Nazmul Islam, A. M. Kafil Uddin, Mohammad Adil Hossain, Talha Bin Emran, Jesus Simal-Gandara

**Affiliations:** 1 Department of Pharmacy, International Islamic University Chittagong, Chittagong 4318, Bangladesh; abutayab.me@gmail.com (M.A.T.); ashfak4u_ctg@yahoo.com (K.A.A.C.); faisalahammed855@gmail.com (M.J.); mail2babor@gmail.com (S.M.T.); mostafa@pharm.iiuc.ac.bd (A.T.M.M.K.); kafilprincebd@gmail.com (A.M.K.U.); adilhossain532@gmail.com (M.A.H.); 2 Department of Pharmacy, BGC Trust University Bangladesh, Chittagong 4381, Bangladesh; 3 Nutrition and Bromatology Group, Department of Analytical and Food Chemistry, Faculty of Food Science and Technology, University of Vigo—Ourense Campus, E32004 Ourense, Spain

**Keywords:** *Woodfordia fruticosa*, antioxidants, oxidative stress, bioactive molecules, medicinal plants, phytochemical screening, molecular docking

## Abstract

Dhaiphul (*Woodfordia fruticosa*) is a frequently demanded plant in South-East Asian regions for its diverse medicinal values. This study was proposed to examine antioxidant, antidiabetic, and antidepressant potentials of methanol extract of *W. fruticosa* leaves (MEWF) and its derived n-hexane (NHFMEWF) and ethyl acetate (EAFMEWF) fractions through in vitro, in vivo, and computational models. Among test samples, MEWF and EAFMEWF contained the highest phenolic content and showed maximal antioxidant activity in DPPH radical scavenging and ferric reducing power assays. In comparison, NHFMEWF possessed maximum flavonoid content and a significantly potent α-amylase inhibitory profile comparable with positive control acarbose. In animal models of depression (forced swimming and tail suspension test), EAFMEWF and NHFMEWF demonstrated a dose-dependent antidepressant-like effect; explicitly, the depressive-like behaviors significantly declined in EAFMEWF-treated dosing groups in contrast to the control group. In the computational analysis, previously isolated flavonoid compounds from Dhaiphul leaves manifested potent binding affinity against several key therapeutic target proteins of diabetes and depressive disorders including α-amylase, serotonin transporter, dopamine transporter, and neuronal nitric oxide synthase with varying pharmacokinetics and toxicity profiles. This research’s outcomes may provide potential dietary supplements for mitigating hyperglycemia, cellular toxicity, and depressive disorder.

## 1. Introduction

Plants and plant extracts provide healthcare for more than three-quarters of the world’s population [1]. They have long been imitative sources of medicine, and the discovery of many of the medicines currently available is somehow (directly or indirectly) linked to them [2]. The World Health Organisation (WHO) data suggested that more than 30% of all plant species have been used for therapeutic purposes at one time or another [1]. Plant-based treatments are becoming progressively prevalent in developed and developing countries due to the drawbacks of synthetic medicines [3]. Dhaiphul [*Woodfordia fruticosa* (L.) Kurz.] is a straggling leafy shrub, belonging to the Lythraceae family [4]. The plant has been recommended as traditional medicine by numerous experts in several regions of South-East Asia [5,6]. In India and Nepal, *W. fruticosa* leaves are used as folk medicines to relieve fever in combination with sugar and dried ginger, and also applied for the management of sitz bath, leucorrhoea, piles etc. [6,7]. It has multiple ethnobotanical applications such as healing bowel disorders, ulcers, diarrhea, dysentery, and other respiratory illnesses, in addition to curing rheumatism [8]. It contains a rich amount of phenolics, especially hydrolyzable tannins and flavonoids, as well as other minor non-phenolic constituents like steroids and triterpenoids [6]. Early experimental studies have demonstrated that *W. fruticosa* leaves extract possesses multiple pharmacological activities including antibacterial [9,10], antioxidant [11], hypoglycemic [12,13], and anti-ulcer activities [14]. However, its α-amylase inhibitory and neuropharmacological potentials were seldom explored.

Oxygen-free radicals or reactive oxygen species (ROS) are continuously generated in our body by endogenous reactions or by interaction with exogenous sources [15]. In biological systems, ROS are proposed to play a binary role as species that can be either detrimental or favorable [16]. As the process of ROS is engaged at low and regulated levels, crucial physiological tasks are facilitated such as signaling cascades and control of cellular processes, including growth, apoptosis, differentiation, proliferation, cytoskeletal regulation, etc. [17]. However, an increased ROS level can contribute to the inconsistency in cellular oxidation state, redox equilibrium, and subsequently can lead to the development of oxidative stress (OS) [18,19]. Persistent and accumulative OS provokes potentially harmful alterations in a diverse range of macromolecular components including DNA, proteins, and lipids [20]. Rich circumstantial evidence demonstrates that OS progressively contributes to the pathogenesis of major chronic diseases, including diabetes, cancer, neurodegenerative, cardiovascular, and pulmonary disease [21]. Antioxidants, a group of compounds, impede autoxidation by blocking the generation of free radicals or by disturbing the dissemination of free radicals through a range of mechanisms [22]. They help to prevent or reduce oxidative damage to cell macromolecules resulting from either a threatening xenobiotic or a changed pathological condition (diabetes) [23]. Targeting OS or enhancing endogenous amounts of antioxidants by supplementing antioxidants is expected to have a protective impact on the treatment of many disorders including diabetes and neurodegenerative diseases [24].

Type 2 diabetes mellitus (DM2) is an intricate metabolic disorder of wide-ranging etiology [25]. An interaction among biological, behavioral, and environmental risk factors is proposed to play a pivotal role in the pathogenesis of DM2 [26]. It is the most prevalent form of diabetes compared to type 1 or gestational diabetes which is accountable for over 90 percent of all cases [27]. DM2 is a disease affecting over 400 million individuals worldwide and the prevalence is anticipated to increase twofold within the following two decades [28]. It is characterized by elevated hyperinsulinemia, insulin resistance, and dysfunctional pancreatic β-cell, with up to 50% β-cell loss at diagnosis [29]. These pathophysiological alterations lead to a raised blood glucose level (hyperglycemia) which is the primary biochemical characteristic of DM2. Persistent hyperglycemia causes oxidative and nitrosative stress, activates pathways involved in inflammation, impairs endothelial functioning, and subsequently contributes to the development of microvascular (neuropathy, nephropathy, retinopathy, etc.) and macrovascular (cardiovascular comorbidities) complications that are found to be the principal causes of morbidity and mortality due to DM2 [27,30]. Diverse classes of antihyperglycemic medications are being used which address different aspects of the DM2 pathogenesis through a variety of actions, but these interventions differ in terms of effectiveness, convenience, cost, and harms [30]. The significance of glycemic management preventing the complications, morbidity, and mortality associated with DM2 has led to intensive research on improved antidiabetic therapies [31]. In DM2, chronic hyperglycemia is expressed in two indexes, i.e., fasting and postprandial blood glucose levels [32]. Increased postprandial blood glucose level or postprandial hyperglycemia (PPHG) is considered to be an important factor in the initiation and development of DM2 [32,33]. Controlling PPHG is proposed as an important therapeutic approach in the management of DM2. This approach is achieved by delaying glucose absorption through intestinal epithelium by inhibiting the carbohydrate-hydrolyzing enzymes such as α-amylase and α-glucosidase in the digestive tract [34,35,36]. Acarbose, voglibose, and miglitol are frequently available DM2 drugs of synthetic origin which control PPHG by suppressing these enzymes [37]. However, these inhibitors are associated with unfavorable side effects because they are non-specific in targeting different enzymes [38,39,40,41,42]. So, the exploration for safer and side-effect-free inhibitors has been aimed towards natural sources, mainly medicinal plants [43]. Phytochemicals and secondary plant metabolites such as phenolic compounds, flavonoids, proanthocyanidins, etc., have been reported to possess amylase inhibitory potentials [40]. Unlike conventional synthetic drugs, these are usually considered safe, harmless, and devoid of unfavorable effects such as cardiovascular and gastrointestinal complications [44]. Therefore, plant-based inhibitors can provide an appealing strategy for the successful management of PPHG in DM2 patients [45].

Major depressive disorder (MDD) is a prevalent, heterogeneous psychiatric disorder characterized by depressive mood, anhedonia, and impaired cognitive function [46]. It is the world’s leading cause of disability, affecting 300 million individuals globally, and is associated with nearly 800,000 suicide cases each year [47]. The neurobiology behind depression still has not been completely established but is claimed to be the consequence of cellular and molecular anomalies that interfere with genetic and environmental factors [48]. The monoamine hypothesis, which has been a core issue of research in the area of pathophysiology and pharmacotherapy for depression, demonstrates that depression may emerge as a consequence of loss of the levels of monoamines, i.e., serotonin/5-hydroxytryptamine (5-HT), norepinephrine, and dopamine in the brain. Most of the antidepressant therapy currently available was designed based on this principle [49]. Other potential etiologies of depression include dysfunction of the hypothalamic–pituitary–adrenal axis, neurogenesis, defects of the second messenger pathway, elevated levels of inflammatory cytokines, and corticotrophin-releasing factor [50]. Despite the availability of various categories of antidepressant drugs for the treatment of depression, the complete relief of depressive symptoms remained elusive due to their severe drawbacks, i.e., poor clinical response rate, high relapse risk, treatment resistance, etc. [48,51]. However, recent research on the connection between OS and depression has offered novel insights into the design of interventions to combat this debilitating disorder [52]. Increasing evidence suggests that depression is strongly linked to reduced antioxidant status and the activation of oxidative/nitrosative pathways [53]. The intervention of oxidative and nitrosative stress in MDD is supported by elevated oxidative (such as nitric oxide (NO), malondialdehyde, and 8-hydroxy-2-deoxyguanosine), and nitrosative stress markers in patients with depression, together with reduced amounts of antioxidants (such as vitamin C, vitamin E, and coenzyme Q10) [53,54,55,56,57]. It has also been observed that OS triggers the annihilation of neural cells by reducing the volume of the hippocampus in patients with depression [58]. However, plant-based therapy targeting the relative linkage between oxidative stress and neurodegeneration at the cellular and molecular levels can enhance therapeutic and drug development approaches against neurodegenerative disorders, including depression [59,60].

Past studies revealed that *W. fruticosa* leaves extract can significantly alleviate hyperglycemia in both normo-glycemic and dexamethasone-induced diabetic animal models [12,13], but still an exact mechanism of action of observed antidiabetic effect has not been established yet. After reviewing the literature, it was found that no previous study was carried out to evaluate the in vitro inhibitory effect of *W. fruticosa* leaves on α-amylase. Furthermore, no studies have yet been conducted to establish its antidepressant potential. Therefore, the aim of the study was to evaluate a possible anti-diabetic mechanism of action of *W. fruticosa* leaves (MEWF) and its derived n-hexane (NHFMEWF) and ethyl acetate fraction (EAFMEWF) to explore their inhibitory effect on α-amylase as well as to ascertain polyphenolic contents and antioxidant potentials through in vitro models, followed by NHFMEWF and EAFMEWF were screened for acute oral toxicity and antidepressant profiles via animal models. Additionally, we employed an in silico molecular docking strategy to study affinities and interactions between previously isolated phenolic compounds from *W. fruticosa* leaves and active site residues of five distinct target proteins of DM2 and depression at the molecular level and followed by pharmacokinetic and toxicity viz. absorption, distribution, metabolism excretion, and toxicity (ADME/Tox) parameters of the compounds were predicted through established tools.

## 2. Materials and Methods

### 2.1. Chemicals and Drugs

Methanol, n-hexane, chloroform, ethyl acetate, Folin–Ciocalteu reagent (FCR), and ascorbic acid (AA) were acquired from Merck (Kolkata, West Bengal, India). α-amylase, starch, iodine, quercetin, aluminum chloride (AlCl_3_), potassium ferricyanide (K_3_[Fe(CN)_6_]), potassium acetate (CH_3_COOK), phosphate buffer, and ferric chloride (FeCl_3_) were procured from Merck (KGaA, Darmstadt, Germany). 2,2-diphenyl-1-picrylhydrazyl (DPPH), trichloro acetic acid (TCA), potassium iodide, and gallic acid (GA) were obtained from Sigma Chemicals Co. (St. Louis, MO, USA). Acarbose and fluoxetine HCl were purchased from Pacific Pharmaceuticals Ltd. and Square Pharmaceuticals Ltd. (Dhaka, Bangladesh), respectively. All the other chemicals used in this experimental work were analytical grade.

### 2.2. Collection and Identification of Plant

Fresh leaves of Dhaiphul (*W. fruticosa*) were collected in September 2019 from lakeside hills of Kaptai, Rangamati district, Chittagong-4500, Bangladesh, and subsequently authenticated by Shaikh Bokhtear Uddin, Professor and Taxonomist, Department of Botany, University of Chittagong, Chittagong-4331, Bangladesh. A voucher specimen (MAT062) of the plant sample has been preserved at the Herbarium of the University of Chittagong (CTGUH) for future reference.

### 2.3. Extraction and Fractionation

The leaves of *W. fruticosa* were washed and cleaned in running tap water and then air-dried for a period of one week at room temperature (25 ± 2 °C, RT). The dried leaves were ground into coarse powder by a mechanical grinder (NOWAKE, Japan) and then 400 g of leaves powder was soaked in 2 L of methanol (95%) for two weeks at RT with occasional stirring. The methanolic mixture was filtered through cotton plugs and then Whatman filter paper No. 1. The resulting mixture was concentrated in a rotary evaporator (Sterilin, UK) at reduced pressure and temperature (50 °C) to get the crude methanol extract (35.60 g; yield 8.9% *w*/*w*).

Fractionation of crude extract was done according to the modified Kupchan solvent-solvent partitioning model [61]. Crude extract (20 g) was suspended into 10% aqueous methanolic solution (crude extract: aqueous methanol = 1: 20 *w*/*v*) and subsequently fractionated with organic solvents (n-hexane, chloroform, ethyl acetate) in order of increasing polarity through a separating funnel. Each fraction was dried by evaporating solvent in a rotary evaporator and the yielded n-hexane (1.76 g) and ethyl acetate (5.64 g) fractions were stored at 4 °C till future analysis.

### 2.4. Quantitative Phytochemical Analysis

#### 2.4.1. Assessment of Total Phenolic Content (TPC)

The TPC of MEWF, NHFMEWF, and EAFMEWF were quantified through employing the Folin–Ciocalteau method. Test samples with polyphenolic contents formed a blue colored complex when reduced by FCR [62]. In short, 0.5 mL (0.5 mg/mL) of each fraction was mixed with 2.5 mL of FCR (10-fold diluted in water) and 2.5 mL of 7.5% (*w*/*v*) sodium carbonate. The mixtures were incubated at 25 °C for 30 min and subsequently, absorbance was read at an optical density (OD) of 720 nm against a blank (methanol) on UV-Visible spectrophotometer (UVmini-1240, Shimadzu, Japan). The same protocol was followed for GA (6.25–100 µg/mL) and a standard calibration curve was constructed by plotting concentration vs. absorbance. Finally, TPC was quantified from that curve as mg of GA equivalent (GAE)/g of dry sample by implementing the Formula (1):

A = (C × V)/m(1)

where A denotes TPC (mg GAE/g of dry sample); C is the concentration of GA (mg/mL) obtained from the calibration curve; V is the extract volume in milliliter (mL) and m stands for weight in gram (g) of the dried plant extract/fractions.

#### 2.4.2. Assessment of Total Flavonoid Content (TFC)

The TFC of the extract was depicted by utilizing the standard calorimetric method [63]. To 0.5 mL of each plant sample (0.5 mg/mL) or quercetin (6.25–200 µg/mL), methanol (3 mL), 10% (*w*/*v*) AlCl_3_ (0.2 mL), 1M CH_3_COOK (0.2 mL) and 5.6 mL of distilled water (DW) were sequentially added. Then, both test and standard mixtures were incubated at 25 °C for 30 min, and afterward, absorbance was determined at OD_415_ nm (optical density) on UV-Visible spectrophotometer against blank (methanol). The TFC of studied samples were computed from the quercetin calibration curve and expressed in mg of quercetin equivalent (QE)/g of dry sample.

### 2.5. Determination of In Vitro Antioxidant Activity

#### 2.5.1. DPPH Free Radical Scavenging Activity

Antioxidant potentials of *W. fruticosa* leaves crude extract and fractions were determined in terms of free radical scavenging capacity by using DPPH as a stable free radical following the protocol designed by Braca, et al. [64]. Just before the test, 0.004% (*w*/*v*) DPPH solution was prepared by methanol in a controlled dark environment. The total assay mixture is composed of 1 mL of the test sample (crude extract/fraction) or standard antioxidant AA at concentrations of 3.125–50 µg/mL and 3 mL of 0.004% DPPH solution. All mixtures were homogenized and incubated at 25 °C for about 30 min in dark. Finally, absorbance was measured at OD_517_ nm (optical density) on UV-Visible spectrophotometer. Methanol and methanolic DPPH solutions served as blank and negative control, respectively. The percentage (%) of free radical-scavenging power was estimated from the following Formula (2):

% scavenging activity = (A_c_ − A_s_)/A_c_ × 100 (2)

where, A_c_ and A_s_ are the absorbance of control and absorbance of the sample (crude extract or fractions or AA) at OD_517_ nm (optical density), respectively.

#### 2.5.2. Ferric Reducing Antioxidant Power (FRAP)

The reducing power was assayed according to a protocol introduced by Oyaizu [65]. In brief, 1 mL of different test samples of *W. fruticosa* leaves at concentrations of 62.5–1000 µg/mL were mixed with the same volume (2.5 mL) of 0.2 M phosphate buffer (pH 6.6) and 1% (*w*/*v*) K_3_[Fe(CN)_6_]. After incubating at 50 °C for a period of 20 min, 2.5 mL of 10% (*w*/*v*) TCA was added to each reaction mixture and immediately centrifuged at 3000 rpm for a period of 10 min. Afterward, supernatant solution (5 mL) from each mixture was taken out and separately mixed with DW (5 mL), 0.1% (*w*/*v*) FeCl_3_ solution (1 mL), and absorbance was instantaneously determined at OD_700_ nm (optical density) through UV-Visible spectrophotometer. AA was served as a reference standard. The increase in the absorbance of the sample with respective concentrations demonstrates the maximum reducing capacity of that sample.

### 2.6. In Vitro Antidiabetic Activity (α-Amylase Inhibition Assay)

The α-amylase inhibitory potential was evaluated through employing the starch-iodine assay method with minor modification, proposed by Xiao, et al. [66]. Prior to testing, α-amylase solution (0.04 mg/mL) was prepared by dissolving 4 mg of α-amylase in 100 mL of 0.02 M sodium phosphate buffer (containing 0.006 M of NaCl, pH 6.9). In brief, 500 mL of test extract/standard (acarbose) at a concentration of 125–1000 µg/mL was added to 500 μL of α-amylase solution (0.04 mg/mL) and incubated at 37 °C for 10 min. Subsequently, 500 μL of 1% (*w*/*v*) soluble starch was added to each test solution and re-incubated at unchanged temperature for 15 min. After re-incubation, the enzymatic reaction was terminated by adding 20 μL of 1M HCl. At last, 100 μL of iodine reagent (0.005 M I_2_ and 0.005 M KI) was added and a change in solution color was noted. The absorbance was read at OD_620_ nm (optical density) on a UV-visible spectrometer. In control, the plant sample was replaced with buffer which represents 100% α-amylase activity. Blank contained only buffer solution instead of the enzyme. The percentage (%) of α-amylase inhibitory activity of test samples and acarbose were calculated by applying the following Equation (3):

% α-amylase inhibition = (A_c_ − A_s_)/A_c_ × 100 (3)

where, A_c_ and A_s_ denote absorbance of control reaction and absorbance of test sample or standard, respectively.

### 2.7. In Vivo Studies

#### 2.7.1. Experimental Animals and Ethical Statements

Adult Swiss albino mice of either sex (weighing between 25 and 30 g) were procured from Pharmacology Laboratory, Jahangirnagar University, Savar-1343, Dhaka. The animals were housed under normal laboratory conditions (temperature: 25 ± 2 °C, relative humidity: 55–60%) in polypropylene cages at 12 h light/dark cycle, and supplied with standard laboratory diet and water ad libitum. The animals were acclimatized to laboratory environments for 10 days before the experiment and all experimental models were executed under noiseless conditions. This experiment was designed and conducted in compliance with the standards and guidelines for the safe use of laboratory animals of the National Institutes of Health (NIH) and the International Council for Laboratory Animal Science (ICLAS). The present research protocol was approved by the Ethics Committee of the Department of Pharmacy, International Islamic University Chittagong, Bangladesh (IIUC/PHARM-AEC-148/13–2019).

#### 2.7.2. Acute Toxicity Studies

The acute oral toxicity study was implemented under standard laboratory conditions following the OECD guidelines [67]. Animals were selected randomly and split into groups (*n* = 5 animals). The control group was administered with vehicle (1% Tween-80 in DW, p.o.) and the test groups were treated with a single dose (100, 200, 400, and 1000 mg/kg body weight, p.o.) of NHFMEWF or EAFMEWF. The animals were then closely monitored for the next 72 h to investigate any behavioral changes, possible signs and symptoms of behavioral toxicity and mortality. The study was conducted under standard laboratory settings, and the mice were kept fasting overnight before the test.

#### 2.7.3. Experimental Design

A total of 30 laboratory animals (15 male and 15 female mice for each test model) were randomly divided into six groups (group I–VI), each of which consists of five animals (*n* = 5). The experimental protocol was designed as follows: Group I or control group received the vehicle (1% Tween-80 in DW, 10 mL/kg b.w., p.o.), group II or positive control group received the standard drug (fluoxetine HCl at 25 mg/kg b.w.,p.o.), and group III and group IV received the NHFMEWF at doses of 100 and 200 mg/kg b.w., p.o., respectively; group V and group VI received the EAFMEWF at doses of 100 and 200 mg/kg b.w., p.o., respectively.

#### 2.7.4. Antidepressant Activity

##### Forced Swimming Test

This test was conducted to screen the antidepressant activity of NHFMEWF and EAFMEWF in mice employing a previously described protocol [68]. The mice were individually placed in an open cylindrical glass compartment (10 × 25 × 19 cm^3^) containing freshwater (25 ± 1 °C) and their mobility was observed for thirty minutes after administration of doses, as mentioned in Section 2.7.3. Then, each mouse was monitored for a period of 6 min, the first 2 min were referred to as the initial adjustment time and the following 4 min were recorded as the period of immobility. The mouse was considered immobile while it remained floating motionlessly or treaded on the water just enough to keep its nose above the water surface and the percent inhibition of immobility was calculated by the following Equation (4):

Inhibition (%) = (A − B)/A ×100(4)

where, A = immobile time in the control group; B = immobile time in the test group.

##### Tail Suspension Test (TST)

This behavioral model for the screening of antidepressant activity in mice was performed using the protocol designed by Steru, et al. [69]. The treatment was followed as mentioned in Section 2.7.3. Thirty minutes after treatment, each mouse was hung at 50 cm above the floor via an adhesive tape placing approximately 1 cm from the tip of the tail to provoke a depressed (immobile) state. The total immobility time of all the groups was recorded for the last 4 min of a total 6 min observation. The percent inhibition was calculated by the following Equation (5):

Inhibition (%) = (A − B)/A × 100(5)

where, A = immobile time in the control group; B = immobile time in the test group.

### 2.8. In Silico Studies

#### 2.8.1. Molecular Docking

Molecular interactions between *W. fruticosa* leaves phytoconstituents and active site of the target proteins was explored through in silico molecular docking approach on Schrödinger Maestro (Maestro, version 11.8, Schrödinger, LLC, New York, NY, USA, 2018) and the resulting ligand–receptor complexes were visualized on BIOVIA Discovery Studio Visualizer v20.0 (BIOVIA, San Diego, CA, USA).

##### Selection of Compounds

**Figure 1:**
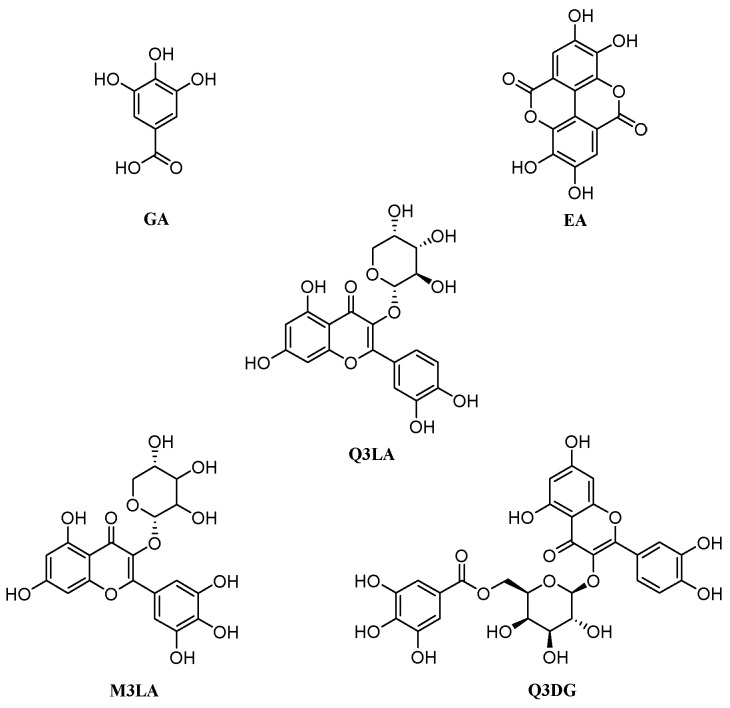
Chemical structures of the potential antidiabetic and antidepressant compounds screened from *W. fruticosa* leaves via computational analysis: GA (gallic acid), EA (ellagic acid), Q3LA (quercetin 3-O-α-L-arabinopyranoside), M3LA (myricetin 3-O-α-L-arabinopyranoside), and Q3DG (quercetin 3-O-(6″-galloyl)-β-D-galactopyranoside).

Leaves of *W. fruticosa* were reported to contain quite several polyphenolic compounds. Kadota, et al. [70] isolated gallic acid and its derivative methyl tri-O-methylgallate, and ellagic acid from the *W. fruticosa* leaves along with six known flavonol glycosides and flavonol glycosides gallates. Therefore, based on the mentioned literature and availability, gallic acid, methyl tri-O-methylgallate, ellagic acid, quercetin 3-O-α-L-arabinopyranoside, myricetin 3-O-α-L-arabinopyranoside, and quercetin 3-O-(6″-galloyl)-β-D-galactopyranoside were enlisted to execute in silico studies (Table 1 and Figure 1).

##### Ligand Preparation

Prior to preparing the ligand, the structures of the enrolled compounds (Table 2) and standard drugs, namely, acarbose, fluoxetine, bupropion, and phenelzine (PubChem CID: 41774, 3386, 444, and 3675, respectively) were retrieved in two-dimensional (2D) SDF format from the PubChem database (https://pubchem.ncbi.nlm.nih.gov (accessed on 20 January 2020)). The compounds were imported in Schrödinger’s Maestro v11.8 and by using Schrödinger’s LigPrep ligand preparation tool the 2D structures were reshaped into energy-minimized three-dimensional (3D) structure under OPLS3e force field. This stage also involved generating a possible ionization state at target pH (7.0 ± 2.0) by Epik (version 4.6), desalting, and generating tautomers [87].

##### Receptor Preparation

The crystal structures of target proteins involved in DM2/depression pathways were downloaded in complex form with ligand(s) from protein data bank (http://www.rcsb.org (accessed on 5 January 2020)) with the following protein data bank (PDB) codes: human pancreatic α-amylase (HPA, PDB ID: 3BAJ) in complex with acarbose [88], serotonin transporter 3 (SERT3, PDB ID: 5I6X) in complex with paroxetine [89], dopamine transporter (DAT, PDB ID: 4M48) in complex with nortriptyline [90], monoamine oxidase A (MAO-A, PDB ID: 2Z5Y) in complex with harmine [91], and neuronal NOS (nNOS, PDB ID: 4UH5) in complex with N1-(5-(2-(6-Amino-4-methylpyridin-2-yl)ethyl)pyridin-3-yl)-N1,N2- dimethylethane-1,2-diamine [92]. On Schrödinger’s Maestro v11.8, the protein preparation wizard module was implemented to fix the PDB protein structure by assigning bond orders, adding hydrogens (H_2_), creating zero-order bonds to metals, creating disulfide bonds, filling missing side chains and loops using Prime v5.4, deleting waters out of 5.00 Å from het groups, and generating het states at pH (7.0 ± 2.0) using Epik v4.6. The structure was further refined by optimizing the orientation of H_2_-bonded groups and finally minimized by the OPLS3e force field. The energy minimization was attained up to RMSD of all heavy atoms expanded 0.3 Å.

##### Grid Generation and Receptor–Ligand Docking

Glide (version 8.1) embedded in Schrödinger Maestro v11.8 (Schrödinger, Inc., New York, NY, USA) was employed to generate the receptor grid and to execute the docking study [93,94]. For each of the resulting proteins from the receptor preparation section, a grid was constructed by maintaining all the parameters at the default setting, i.e., van der Waals scaling factor was kept at 1.00 and charge cutoff at 0.25 subjected to OPLS3e force field. The centroid of the complexed molecule was ascertained as the ligand-binding pocket of the receptor and, after centering it, a cubic box of precise dimension was generated. The ligand box dimension was set to 14 Å × 14 Å × 14 Å to dock the ligand. Afterward, flexible ligand docking was conducted by employing the glide-SP (Glide-Standard Precision) scoring function. Docking calculations were executed under default parameters with no receptor–ligand constraints. The best-docked ligands that possessed maximum favorable binding energetics were ranked by glide-score function with the highest negative docking score. After visualizing the receptor–ligand complex, the molecular interactions were analyzed among studied ligand–receptor complexes.

#### 2.8.2. Predictions of ADME/Tox Profiles

In silico pharmacokinetic, i.e., ADME (absorption, distribution, metabolism, and excretion), properties were predicted through the SwissADME (SIB, Batiment amphipole, Switzerland) web-based tool (http://www.swissadme.ch (accessed on 20 January 2020)) [95]. Physicochemical properties of the selected compounds were predicted by considering Lipinski’s rule of five (RO5) and the number of free rotatable bonds which relates the physicochemical properties of drug candidates to their pharmacokinetic potentials, and it was proposed that compounds are likely to possess higher permeability and absorption characteristics if they meet following parameters: molecular weight (MW) ≤ 500 g/mol), number of H-bond acceptors (HBA) ≤ 10, number of H-bond donors (HBD) ≤ 5, and lipophilicity (Log P) ≤ 5 and number of free rotatable bonds (nRB) ≤ 10 [96]. Pharmacokinetic profiles including different absorption (GI absorption, P-gp substrate) and metabolism (cytochromes P450 enzymes inhibition) parameters were also predicted from the same tool. Toxicity profiles of the selected compounds such as Ames toxicity (mutagenicity), hepatoxicity, and skin sensitization were studied by the pkCSM (Bio21 Institute, Parkville Melbourne, Australia) web server (http://biosig.unimelb.edu.au/pkcsm/prediction (accessed on 15 February 2020)) [97]. Rat acute toxicity LD_50_ (mg/kg) and acute toxicity classification were forecasted via the GUSAR (IBMC, Moscow, Russia) online tool (http://www.way2drug.com/gusar/acutoxpredict.html (accessed on 25 February 2020)) [98]. The toxicity classification was stated in compliance with OECD (Organisation for Economic Cooperation and Development) guidelines for the testing of chemicals [99].

### 2.9. Statistical Analysis

Each in vitro assay was executed in triplicate (*n* = 3), and for α-amylase inhibition and DPPH radical scavenging assay, dose–response curves were constructed by plotting % α-amylase inhibition versus concentration (µg/mL) and percent (%) scavenging activity versus concentration (µg/mL), respectively, and afterward, values of half-maximal inhibitory concentration (IC_50_) were ascertained through non-linear regression analysis using GraphPad Prism version 8.0.2 for Windows (GraphPad Software, San Diego, CA, USA). The result is exhibited as mean ± SEM (standard error means). The differences among/between test groups and control group were analyzed by one-way analysis of variance (ANOVA) followed by implementing Tukey’s HSD (honest significant difference) or Dunnett’s multiple comparison *post hoc* test with a significance level (α) = 0.01. All statistical analyses were accomplished via IBM SPSS software for Windows, version 26.0 (IBM Corp., Armonk, NY, USA).

## 3. Results

### 3.1. Total Phenolic and Flavonoid Contents

The TPC and TFC were quantified from the GA calibration curve (y = 0.008x + 0.01, R^2^ = 0.998) and quercetin calibration curve (y = 0.003x + 0.024, R^2^ = 0.995), respectively. As presented in Table 3, MEWF and EAFMEWF were ascertained to be contain the maximum amount of TPC (254.42 ± 1.53 and 238.04 ± 1.01 mg of GAE/g of dry sample, respectively) whereas TFC ascertained in NHFMEWF was found to be the highest (371.10 ± 1.99 mg of QE/gm of dry sample). Considering TPC and TFC; MEWF, NHFMEWF and EAFMEWF can be ranked as follows: For TPC: MEWF > EAFMEWF > NHFMEWF; For TFC: NHFMEWF > EAFMEWF > MEWF.

### 3.2. In Vitro Antioxidant Activity

#### 3.2.1. DPPH Radical Scavenging Activity

**Figure 2:**
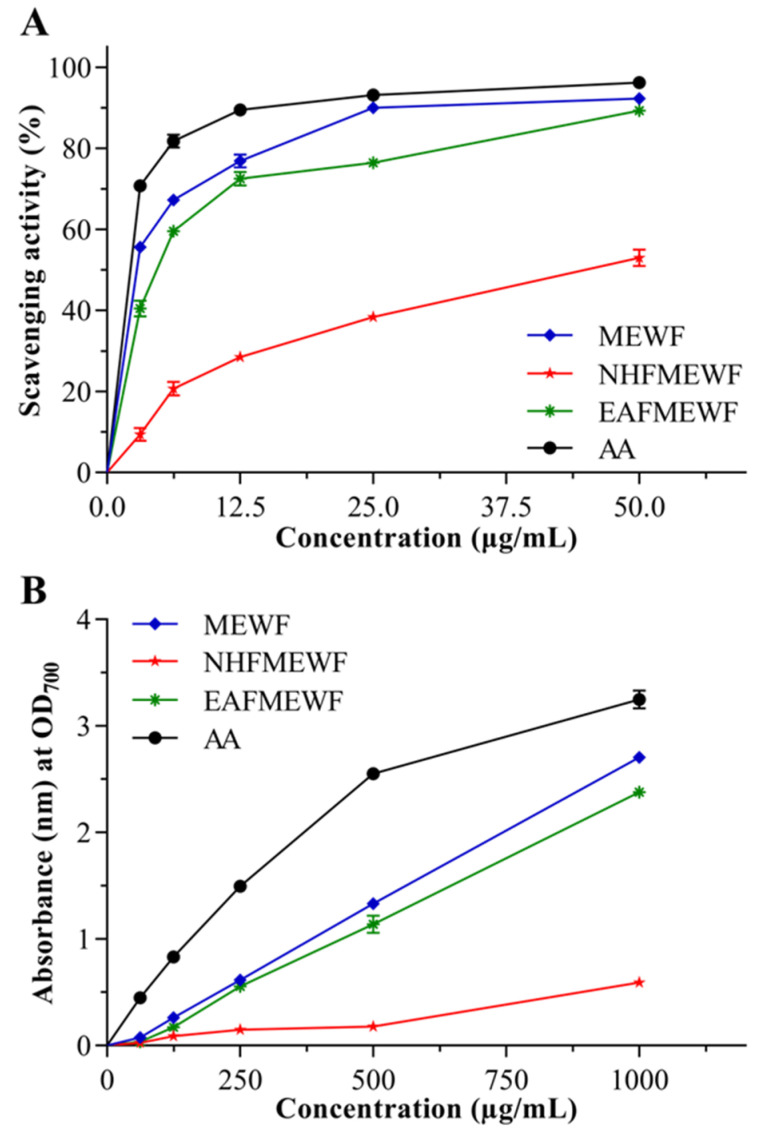
(**A**) Percentage of DPPH free radical scavenging activity, and (**B**) ferric reducing antioxidant power of methanol extract of the *W. fruticosa* leaves and its derived different fractions at five different concentrations (µg/mL) compared to reference antioxidant (ascorbic acid). Values are figured as mean ± SEM (*n* = 3). MEWF, methanol extract of *W. fruticosa* leaves; NHFMEWF, n-hexane fraction of methanol extract of *W. fruticosa* leaves; EAFMEWF, ethyl acetate fraction of methanol extract of *W. fruticosa* leaves; AA, ascorbic acid.

The antioxidant power of MEWF, NHFMEWF, and EAFMEWF is mentioned, in percentage form, in Figure 2A, concerning the ability to scavenge DPPH free radical. MEWF manifested the strongest radical scavenging activity (IC_50_ = 1.86 ± 0.16 µg/mL) compared to EAFMEWF and NHFMEWF, which is comparable with the positive control AA (IC_50_ = 0.22 ± 0.02 µg/mL). The IC_50_ values of EAFMEWF and NHFMEWF were assessed to be 4.30 ± 0.28 µg/mL and 47.03 ± 0.57 µg/mL, respectively (Table 3).

#### 3.2.2. Ferric Reducing Power Capacity

Figure 2B represents the ability of MEWF, NHFMEWF, and EAFMEWF to reduce Fe^3+^ to Fe^2+^ ions. Dose–response relationships were seen in the case of all test samples and AA (positive control) where the highest absorbance (nm) suggests the strongest ability to reduce Fe^3+^ ions. In comparison to ascorbic acid, MEWF was found to possess potent reducing power among all test samples, followed by EAFMEWF, while NHFMEWF was least strong. At 1000 µg/mL concentration, the absorbance of ascorbic acid, MEWF, EAFMEWF and NHFMEWF were measured to be 3.250 ± 0.089, 2.705 ± 0.058, 2.380 ± 0.046, and 0.591 ± 0.051, respectively, at OD_700_ nm.

### 3.3. In Vitro Antidiabetic Activity (α-Amylase Inhibitory Activity)

**Figure 3:**
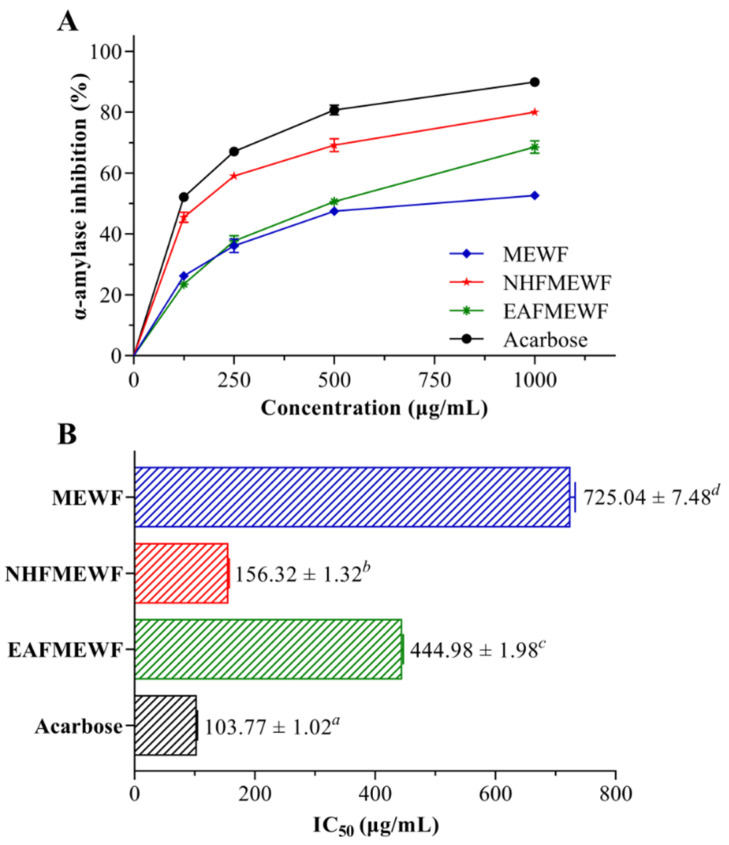
Anti-α-amylase activities of methanol leaves extract of *W. fruticosa* and its different fractions compared with the reference inhibitor acarbose. Values are shown as mean ± SEM (*n* = 3). (**A**) The percentage inhibition of α-amylase activity at four different concentrations (µg/mL). (**B**) IC_50_ values (µg/mL) for α-amylase activity. Values marked with different superscript letters (*a*, *b*, *c*, *d*) in different columns signify statistical differences at *p* < 0.01 (Tukey’s HSD test). MEWF, methanol extract of *W. fruticosa* leaves; NHFMEWF, n-hexane fraction of methanol extract of *W. fruticosa* leaves; EAFMEWF, ethyl acetate fraction of methanol extract of *W. fruticosa* leaves.

In the starch-iodine method, degradation of starch in reaction mixtures was visually perceived based on the change in the solution color and latterly spectrophotometrically assessed at OD_620_ nm. In the presence of potential inhibitors in the experimental samples, the starch did not degrade in the enzyme-containing assay mixtures and gave rise to a dark-blue color complex. Additionally, yellow color and brownish color were observed in the absence of inhibitors and the presence of partial inhibitors, respectively, indicating complete hydrolyzation of starch and partial hydrolyzation of starch, respectively, by α-amylase. The anti-α-amylase potential of crude extract and fractions of *W. fruticosa* leaves is displayed in Figure 3. Among studied test samples, NHFMEWF possessed the most potent enzyme inhibitory activity (IC_50_ = 156.32 ± 1.32 µg/mL) which is significant (*p* < 0.01) when compared to standard inhibitor acarbose (IC_50_ = 103.77 ± 1.02 µg/mL). EAFMEWF exhibited moderate anti-α-amylase activity (IC_50_ = 444.98 ± 1.98 µg/mL) whereas MEWF was found to possess minimum inhibitory activity (IC_50_ = 725.04 ± 7.48 µg/mL).

### 3.4. In Vivo Studies

#### 3.4.1. Acute Toxicity Study

The oral acute toxicity profiles of NHFMEWF and EAFMEWF were studied on Swiss albino mice. NHFMEWF or EAFMEWF, at a single oral dose of 100–1000 mg/kg b.w., did not produce any signs of toxicity and mortality in the experimental animals during 72 h of post-treatment. This study suggested that NHFMEWF and EAFMEWF possessed an LD_50_ value greater than 1000 mg/kg. Therefore, based on this observation, dose levels of 100 and 200 mg/kg b.w. were chosen for the current experiment.

#### 3.4.2. Antidepressant Activity

##### Forced Swimming Test (FST)

**Figure 4:**
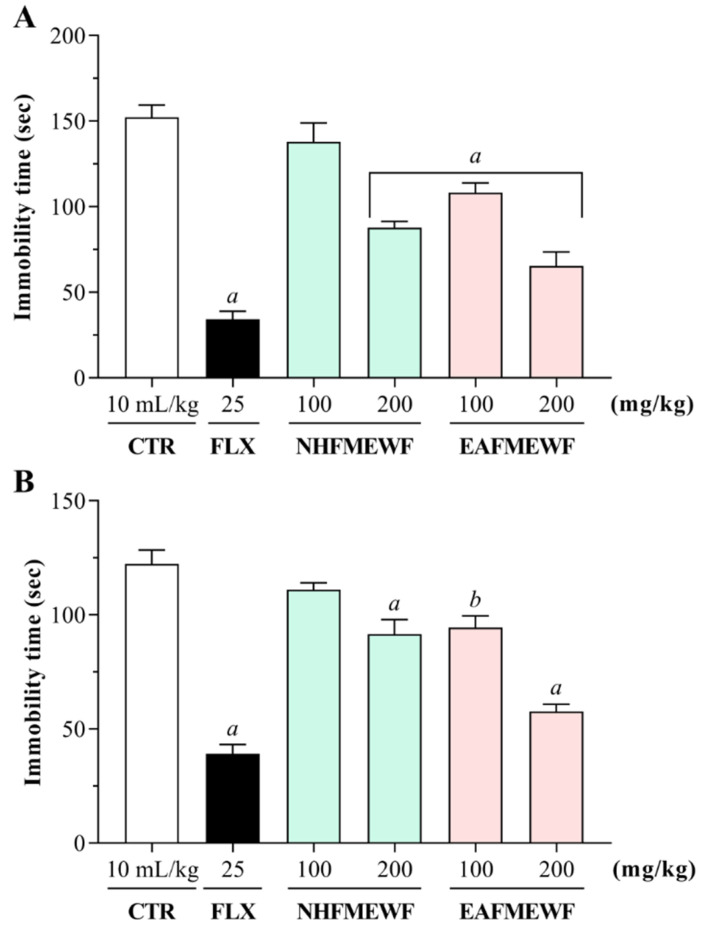
Effect of oral treatment of NHFMEWF and EAFMEWF in mice on (**A**) forced swimming test, and (**B**) tail suspension test, against vehicle-treated (control) and fluoxetine-treated (reference antidepressant) group. NHFMEWF (100 and 200 mg/kg), EAFMEWF (100 and 200 mg/kg), and fluoxetine (25 mg/kg) were administered 30 min prior to the tests. Each column represents mean ± SEM (*n* = 5 animals). *^b^ p* < 0.01, *^a^ p* < 0.001 vs. control group (one way ANOVA followed by Dunnett’s Test). CTR, control; FLX, fluoxetine HCl; NHFMEWF, n-hexane fraction of methanol extract of *W. fruticosa* leaves; EAFMEWF, ethyl acetate fraction of methanol extract of *W. fruticosa* leaves.

The possible antidepressant effects of NHFMEWF and EAFMEWF in the FST have been shown in Figure 4A. In this behavioral model, mice treated with NHFMEWF and EAFMEWF at doses of 100 and 200 mg/kg demonstrated a dose-dependent inhibition of immobility. In FST, 100 and 200 mg/kg dose of NHFMEWF and EAFMEWF exhibited 57.02% (65.47 ± 8.15 s; *p* < 0.001), 7.88% (140.38 ± 3.15 s; *p* = 0.49), 28.98% (108.18 ± 5.72 s; *p* < 0.001) and 42.42% (87.71 ± 3.71 s; *p* < 0.001) inhibition of immobility, respectively, compared to the control group. Besides, mice treated with the standard drug (fluoxetine HCl at 25 mg/kg; p.o.) showed a maximum reduction of 77.57% (34.17 ± 4.73 s; *p* < 0.001 vs. control group) in the duration of immobility.

##### Tail Suspension Test (TST)

The effects of NHFMEWF and EAFMEWF on the immobility time of mice in the TST have been shown in Figure 4B. As seen in the figure, administration of NHFMEWF and EAFMEWF by oral route at doses of 100 or 200 mg/kg manifested a dose-dependent decrease in the immobility time of the experimental animals. Among the sample therapies, the group treated with EAFMEWF at a dose of 200 mg/kg reported the highest and most significant antidepressant effect compared to the control group. EAFMEWF (200 mg/kg) reduced the immobility time by 52.79% (57.74 ± 3.03 s; *p* < 0.001). Groups treated with NHFMEWF (200 mg/kg) and EAFMEWF (100 mg/kg) exhibited inhibitions of immobility time by 25.17% (91.52 ± 6.38 s; *p* < 0.001 vs. control), and 22.75% (94.48 ± 5.03 s; *p* < 0.01 vs. control), respectively. However, NHFMEWF at 100 mg/kg dose did not demonstrate a significant reduction (*p* = 0.35 vs. control group) in immobility time. As anticipated, standard drug fluoxetine at a dose of 25 mg/kg (p.o.) significantly alleviated the immobility time in mice by 67.97% (39.17 ± 3.99 s; *p* < 0.001) compared to control.

### 3.5. In Silico Studies

#### 3.5.1. Molecular Docking

Recognizing the abundant presence of polyphenols in the studied extract/fractions of *W. fruticosa* leaves, in the current study, involved a molecular docking approach wherein six previously isolated polyphenolic compounds were docked against several target proteins of DM2 (HPA, PDB ID: 3BAJ) and MDD (SERT3, PDB ID: 5I6X; DAT, PDB ID: 4M48; MAO-A, PDB ID: 2Z5Y; nNOS, PDB ID: 4UH5). The docking scores and primary binding interactions of the compounds to the active site of the respective protein have been categorized in Table 4 and Table S1, respectively.

**Figure 5:**
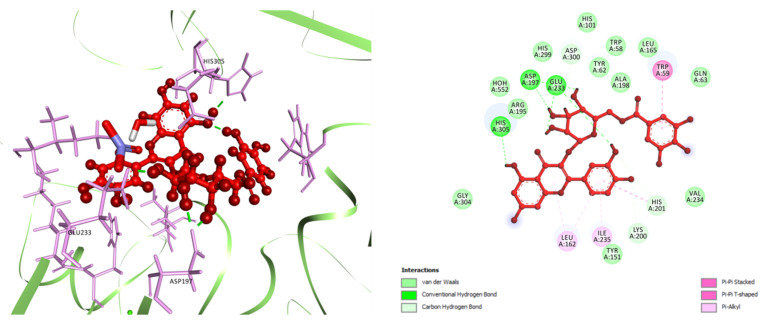
Best ranked docking pose (left) and 2D visualization of the interactions (right) of Q3DG in the catalytic pocket of HPA (PDB ID: 3BAJ). Q3DG, quercetin 3-O-(6″-galloyl)-β-D-galactopyranoside.

In docking analysis for DM2, among six selected compounds, Q3DG manifested the strongest affinity to bind against HPA (PDB ID: 3BAJ) with a docking score of −7.41 kcal/mol, followed by Q3LA and M3LA (docking score −6.84 and −6.105 kcal/mol, respectively). GA, MTMG, EA, and acarbose (standard anti-α-amylase drug) managed to achieve docking scores of −5.136, −3.429, −5.08, and −7.752 kcal/mol, respectively (Table 4). In terms of docking score, the order of ranking of the six studied compounds and acarbose are as follows: acarbose > Q3DG > Q3LA > M3LA > GA > EA > MTMG. Analysis of ligand–receptor complexes uncovered varied binding interactions between the target enzyme and the studied compound (Table S1, Figure 5 and Figure S1). The best-scored Q3DG bound to HPA through five H-bond interactions with Asp197 (two interactions), Glu233 (two interactions) and His305, three carbon-hydrogen (C-H) bond with Lys200, His201 and Asp300, one pi-pi stacked bond with Trp59, one pi-pi t-shaped bond with His201, and three pi-alkyl bonds with Leu162 (two interactions) and Ile235 (Figure 5). The following best-docked compounds: Q3LA manifested affinity against HPA via the formation of six H-bond interactions with residue Trp59, Asp197 (two interactions), His 299, Asp300 and His305, and two C-H bonds with Glu233 and Asp300; M3LA interacted by forming nine H-bond interactions with Thr163, Arg195, Asp197, Lys200, Glu233, His 299, Asp300 and His305 (two interactions), three C-H bond interactions with Ala198 and Asp300 (two interactions), one pi-donor H-bond with Tyr62, one pi-pi stacked bond with Tyr151, one pi-pi t-shaped bond with His201, one pi-sigma bond with Ile235, and two pi-alkyl bonds with Leu162 and Lys200 (Figure S1). GA, EA, and MTMG possessed affinity towards the target enzyme (HPA) primarily by establishing two, four and one H-bond interactions and four, three and nine hydrophobic interactions, respectively (Table S1). Additionally, acarbose interacted with the active site of HPA via the formation of ten H-bonds with Trp59, Thr163, Arg195, Lys200, Glu233, Glu240, Asp300 (two interactions) and His305 (two interactions), one electrostatic interaction with Asp 300 (attractive charge), five C-H bonds with Tyr151, Asp197, Glu233 (two interactions) and Asp300, two alkyl bonds with Leu162 and Leu165, and one pi-alkyl bond with His101 (Figure S1).

**Figure 6:**
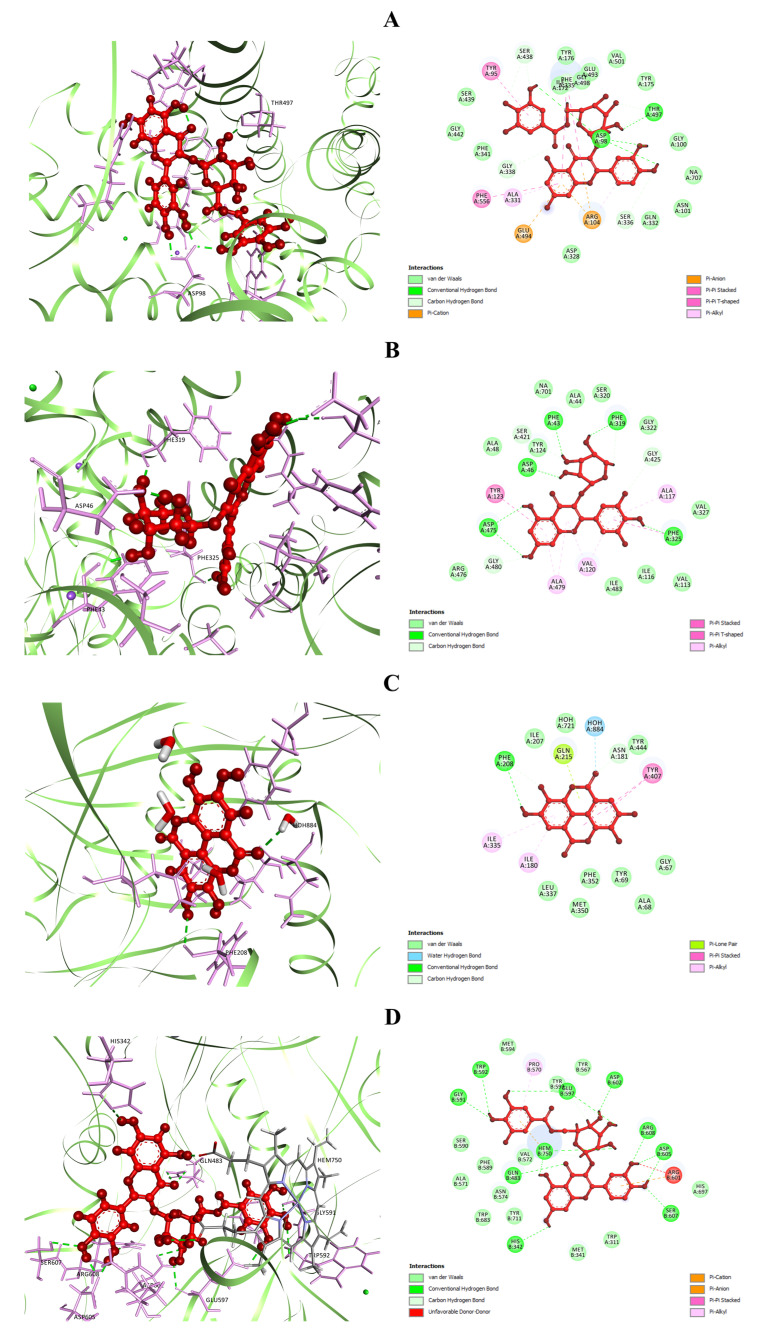
Best ranked docking pose (left), and 2D visualization of the interactions (right) of (**A**) Q3DG, (**B**) M3LA, (**C**) EA, and (**D**) Q3DG with active site residues of SERT3 (PDB ID: 5I6X), DAT (PDB ID: 4M48), MAO-A (PDB ID: 2Z5Y), and nNOS (PDB ID: 4UH5), respectively.

The selected compounds were then docked against SERT3 (PDB ID: 5I6X) to delineate the potential mechanism behind the antidepressant-like activity of *W. fruticosa* leaves. Analysis of docking scores revealed that from the studied compounds, Q3DG possessed maximum binding energetics against the target protein (SERT3) with a docking score of −8.678 kcal/mol, followed by Q3LA (docking score −8.398 kcal/mol), M3LA (docking score −7.378 kcal/mol), EA (docking score −6.415 kcal/mol), GA (docking score −5.859 kcal/mol), and MTMG (docking score −4.782 kcal/mol). Fluoxetine showed a docking score of −9.426 kcal/mol (Table 4). Analysis of the orientation of studied compounds at the active site of SERT3 exposed a quite number of binding interactions between ligands and the target protein (Table S1, Figure 6A and Figure S2). The best-docked Q3DG interacted with the active site of SERT3 via the formation of four H-bonds with Asp98 (three interactions) and Thr497, six C-H bonds with Ser336, Gly338, Ser438 (two interactions) and Thr497 (two interactions), three electrostatic interactions with Arg104 (two pi-cation interactions) and Glu494 (pi-anion interactions), three pi-pi stacked bonds to Phe335 (two interactions) and Phe556, one pi-pi t-shaped bond to Tyr95, and two pi-alkyl bonds to Arg104 and Ala331 (Figure 6A). Q3LA manifested affinity against the same target protein (SERT3) through two H-bond interactions with Asp98 and Phe335, three C-H bond interactions with Asp98, Gly338 and Ser438, one pi-donor H-bond interaction with Tyr176, two pi-pi t-shaped bonds to Tyr176, four pi-alkyl bonds to Ala169, Ile172 (two interactions) and Ala173, and one pi-lone pair bond to Tyr176 (Figure S2). M3LA, EA, and GA interacted with SERT3 mainly through five, three, and four H-bond interactions, respectively, and several hydrophobic interactions. MTMG did not exhibit any H-bond interaction but revealed five hydrophobic interactions (Table S1). Fluoxetine bound to SERT3 through one H-bond interaction with Tyr95, eight C-H bond interactions with Ala96, Asp98 (two interactions), Ala173, Ser336, Leu337, Ser439 and Gly442, two electrostatic interactions with Asp98 (attractive charge) and Tyr95 (pi-cation charge), two pi-pi t-shaped bonds to Tyr176 and Phe441, two alkyl bonds to Ile172 and Ala173, three pi-alkyl bonds to Ile172 (two interactions) and Val501, and six halogen (fluorine) interactions with Ala169, Ile172, Ala173, Ser439 and Gly442 (two interactions) (Figure S2).

The docking scores of the selected polyphenolic compounds against DAT (PDB ID: 4M48) are displayed in Table 4. The docking analysis demonstrated that M3LA and Q3DG possess the greatest binding affinity against DAT with the highest docking scores of −10.796 and −10.62 kcal/mol, followed by Q3LA (docking score −9.794 kcal/mol) and EA (docking score −7.658 kcal/mol). The docking scores for GA and MTMG were assessed to be −4.838 and −6.015 kcal/mol, respectively, whereas bupropion, a norepinephrine/dopamine reuptake inhibitor, manifested a docking score of −7.159 kcal/mol. The rankings for the selected compounds and bupropion are as follows concerning the docking score: M3LA > Q3DG > Q3LA > EA > bupropion > MTMG > GA. An in-depth study of the ligand–receptor complexes revealed that the highest-scored M3LA exerts affinity at the active site of DAT through the formation of six H-bond interactions with Phe43, Asp46, Phe319, Phe325 and Asp475 (two interactions), three C-H bond interactions with Tyr124, Phe319 and Gly425, one pi-pi stacked bonds with Phe325, one pi-pi t-shaped bond with Tyr123, and five pi-alkyl bonds with Ala117, Val120 (two interactions) and Ala479 (two interactions) (Figure 6B). Q3DG interacted forming eight H-bonds with Trp51, Tyr123, Phe325, Asp475 (four interactions) and Arg476, one electrostatic interaction with Asp475 (pi-anion), two pi-pi stacked bonds with Phe325, five pi-alkyl bonds with Ala117, Val120 (two interactions) and Ala479 (two interactions). Q3LA bound to the same target protein (DAT) via the formation of four H-bond interactions with Phe43, Phe325, Asp475 (two interactions), three C-H bonds with Phe319, Ser421 and Gly480, one pi-pi stacked bond with Phe325, one pi-pi t-shaped bond with Tyr123, and seven pi-alkyl bonds to Ala117, Val120 (two interactions), Ala479 (three interactions) and Ile483; followed by EA bound by forming two H-bond interactions with Asp46 and Phe325, one C-H bond interaction with Ser421, three pi-pi stacked bonds with Phe325, one pi-pi t-shaped bonds with Tyr124, and four pi-alkyl interactions with Val120 (three interactions) and Ala479 (pi-alkyl) (Figure S3). MTMG mainly interacted with the active site residues of DAT through thirteen hydrophobic interactions, while GA interacted primarily by forming three hydrogen bond interactions and one hydrophobic interaction (Table S1). Additionally, bupropion manifested affinity against DAT via the formation of one H-bond interaction with Phe319, one C-H bond interaction with Phe319, one salt-bridge to Asp46, one pi-pi stacked bond with Phe325, two alkyl bonds with Ala117 and Val120, and three pi-alkyl bonds with Val120, Phe325 and Ala479 (Figure S3).

The docking analysis for antidepressant activity was reinvestigated targeting the MAO-A enzyme (PDB ID: 2Z5Y). EA was analyzed to have the most potent affinity against the active site of MAO-A with a docking score of −7.951 kcal/mol (Table 4), followed by GA and MTMG (docking scores −7.86 and −6.532 kcal/mol, respectively). However, Q3LA, M3LA, and Q3DG did not dock at all. Phenelzine has demonstrated lower binding energetics (docking score −6.782 kcal/mol) compared to EA and GA. The study of the ligand–receptor complexes obtained from the docking study disclosed multiple binding interactions between ligands and receptors (Table S1, Figure 6C and Figure S4). The best docked, EA, bound to the target site of MAO-A through one H-bond interaction with Phe208, two C-H bond interactions with Asn151 and Phe208, one water–hydrogen bond interaction with HOH884, two pi-pi stacked bonds to Tyr407, two pi-alkyl bonds to Ile180 and Ile335, and one pi-lone pair interaction with Gln215 (Figure 6C); followed by GA bound by forming two H-bond interactions with Ala68 and Tyr69, one C-H bond interactions with Gly67, one water–hydrogen bond interaction with HOH884, one electrostatic interaction with Lys305 (attractive charge), and two pi-pi stacked bonds to Tyr407 and Tyr444 (Figure S4). MTMG interacted with the same protein (MAO-A) by establishing five C-H bonds to Gly66, Gly67, Ile180, Asn181 and Gly443, one water–hydrogen bond to HOH884, two pi-pi stacked bonds to Tyr407 and Tyr444, one alkyl bond to Lys305, and five pi-alkyl bonds to Phe352, Tyr407 and Tyr444 (Figure S4). Phenelzine docked to the active site of MAO-A by the formation of three H-bonds with Ile180, Asn181 and Gln215, one C-H bond with Gln215, three pi-alkyl bonds with Ile180, Ile335 and Leu337, and one pi-sulfur bond with Cys323 (Figure S4).

The docking study for antidepressant activity was further conducted targeting the nNOS (PDB ID: 4UH5). From the selected polyphenols, the highest and lowest binding energetics against nNOS were ascertained for Q3DG (docking score −8.449 kcal/mol) and MTMG (docking score −3.699 kcal/mol), respectively. However, GA, EA, Q3LA, and M3LA manifested docking scores of −4.688, −5.329, −5.378, and −5.912 kcal/mol, respectively (Table 4). The rankings for the studied compounds are as follows concerning the docking performance against nNOS: Q3DG > M3LA > Q3LA > EA > GA > MTMG. Analysis of the position of docked compounds at the active site of nNOS has demonstrated quite a variety of binding interactions between ligands and nNOS (Table S1, Figure 6D and Figure S5). The best-scored Q3DG manifested potent affinity at the active site of nNOS by forming thirteen H-bond interactions with His342, Gln483, Gly591, Trp592, Glu597 (two interactions), Asp602, Asp605 (two interactions), Ser607, Arg608 and Hem750 (two interactions), four C-H bonds with Glu597 (two interactions) and Hem750 (two interactions), two electrostatic interactions with Arg601 (pi-cation) and Hem750 (pi-anion), one pi-pi stacked bond with Hem750, and one pi-alkyl bond with Pro570 (Figure 6D). M3LA bound to nNOS via ten H-bond interactions with Gln483, Asn574, Tyr593 (two interactions), Glu597 (two interactions), Arg601, Asp602, Asp605 and Hem750, one C-H bond interaction with Hem750, one pi-donor H-bond interaction with Asn574, and one electrostatic interaction with Hem750 (pi-anion) (Figure S5). Q3LA interacted with the same target (nNOS), through the formation of eight H-bonds with Trp311 (two interactions), Gln483, Ser607, Asp714 (two interactions), and Hem750 (two interactions), followed by EA, which interacted through six H-bond interactions with Gln483, Tyr593, Asp602 (two interactions) and Hem750 (two interactions), and one hydrophobic interaction with Val572 (pi-alkyl) (Figure S5). Besides, GA and MTMG bound mainly by forming four and one H-bond interactions, and one and seven hydrophobic interactions, respectively, with the active site residues of nNOS (Table S1).

#### 3.5.2. ADME/Tox Profiles

The drug-likeness of the selected isolated compounds from *W. fruticosa* leaves were studied concerning the physicochemical parameters stated in Lipinski’s RO5 viz. molecular weight, number of H-bond acceptors, number of H-bond donors, lipophilicity, and number of rotatable bonds. As shown in Table 5, all of the physicochemical properties of compounds GA, MTMG and EA were within the acceptable range mentioned in RO5. Q3LA and M3LA violated two rules (HBA and HBD) among RO5, whereas compound Q3DG was ascertained to violate three rules (MW, HBA, and HBD).

Table 6 discloses the pharmacokinetics, i.e., absorption and metabolism profiles of the five best-docked compounds. GA, MTMG, and EA were found to possess high GI absorption; conversely, compounds Q3LA, M3LA, and Q3DG were seen to have low GI absorption. All compounds were found to be non-P-gp substrate, and excluding GA and EA, the other compounds were non-inhibitors of all CYP (cytochrome P450) enzymes. The report of the toxicity profiles of six compounds exhibited in Table 6. The compounds were found to have a negative Ames toxicity (excluding MTMG), hepatoxicity, and skin sensitization profile. The LD_50_ (mg/kg) values for rat oral acute toxicity were reckoned to be between 1606 and 3501 mg/kg. Additionally, GA and EA were demonstrated to have an acute toxicity level of Class 4 (slightly toxic), whereas MTMG, Q3LA, M3LA, and Q3DG were deduced to have an acute toxicity level of Class 5 (non-toxic). Additionally, the toxicity level of all of the selected compounds falls within the OECD applicability domain (AD) of models (Table 7).

## 4. Discussion

The current research was hypothesized to investigate the methanol extract of *W. fruticosa* leaves and their n-hexane (NHFMEWF) and ethyl acetate (EAFMEWF) fraction as a possible inhibitor of the HPA enzyme, as well as to ascertain their polyphenolic contents (TPC and TFC) and antioxidant activities. An in vivo approach was employed to demonstrate the potential antidepressant-like activity of two extracted fractions (NHFMEWF and EAFMEWF). Besides, six isolated polyphenolic compounds from *W. fruticosa* leaves were used to determine the binding affinity to the active site of HPA (target protein for DM2), and SERT3, DAT, MAO-A, nNOS (target proteins for depression) by molecular docking and ADME/Tox profiles.

Polyphenols comprise one of the most abundant and pervasive classes of plant metabolites that provide a broad spectrum of biological activities including antioxidant activity [100,101,102]. Meanwhile, in this study, the quantified polyphenolic contents, namely TPC and TFC, were found to be high in MEWF (254.42 ± 1.53 mg of GAE/gm of dry sample) and NHFMEWF (371.10 ± 1.99 mg of QE/g of dry sample), respectively. The TPC assessed in EAFMEWF (238.04 ± 1.01 mg of GAE/gm of dry sample) was almost close to that of MEWF and it was shown to contain a fair amount of TFC (97.11 ± 0.67 mg of QE/gm of dry sample). The antioxidant properties are typically determined by phenolic constituents [103]. Scientific findings have previously documented that plant phenolic content is predominately liable for their free radical scavenging function and reducing strength [104]. Phenols possess hydroxyl groups that spur their antioxidant’s power through donating hydrogen. Besides, their redox properties empower them to scavenge free radicals and, consequently, enable them to function as a reducing agent [105]. In this testing, the antioxidant capacity of crude extract and various fractions (n-hexane, ethyl acetate) of *W. fruticosa* leaves was interpreted from the DPPH free radical scavenging and ferric power reduction assay. The anti-radical activity of various antioxidants can be easily assessed using DPPH. The introduction of an antioxidant to the DPPH solution reflects a decreased absorbance in proportion to its (antioxidant) concentration and antioxidant potential [106]. In the FRAP assay, the yellow color reaction mixture shifts to green and Prussian blue depending on the reducing capacity of the antioxidants, and the reduction capacity can be ascertained from the absorption of the final reaction at OD_700_ nm [107]. In the current study, both antioxidant assay models (DPPH and FRAP) demonstrated that MEWF is a quite potent antioxidant followed by EAFMEWF; however, NHFMEWF is less potent. Our results disclosed that the test samples (MEWF and EAFMEWF) with high phenol content possess a higher antioxidant potential compared to the test sample (NHFMEWF) with lower phenolic content. From this insight, it may be possible to halt the oxidative progression of numerous diseases like cancers, neurodegenerative and cardiovascular conditions, osteoporosis, and diabetes mellitus [108,109].

Type 2 diabetes mellitus (DM2) has reached an epidemic level, causing severe health and economic losses worldwide. The WHO has listed Diabetes Mellitus as one of four leading non-communicable diseases that needs to draw a serious response from all primary shareholders; it is seen as the third major causal factor for global premature mortality largely due to hyperglycemia [110]. The results have marked OS as playing a crucial role in the pathological process seen in DM2. OS has previously been associated with diabetic complications, and more recent findings have uncovered that in pre-diabetes, OS is also a causative factor in the development of β-cell dysfunction and insulin resistance, two of the defining traits of DM2 [111]. Plant-derived antioxidants are known to be a significant solution in retarding the prognosis of diabetes since they are effective in neutralizing ROS and alleviating OS [112,113]. Therefore, plants possessing a strong enzyme inhibitory and antioxidant potential are considered a probabilistic therapeutic candidate for managing diabetes [114].

The findings of the α-amylase inhibitory study disclosed that the n-hexane fraction of *W. fruticosa* methanolic leaves extract (NHFMEWF) possessed strong α-amylase inhibitory potentials and, in the cases of crude extract (MEWF) and ethyl acetate fraction (EAFMEWF), their α-amylase inhibitory power was found to be mild to moderate compared with acarbose. Considering IC_50_ value, NHFMEWF (156.32 ± 1.32 µg/mL) was estimated to be just around 50% less potent than that of acarbose (103.77 ± 1.02 µg/mL) in inhibiting α-amylase, which was also analyzed to be highly significant (*p* = 0.000063) in contrast to the acarbose. Past evidence suggests that plant-originated bioactive compounds like flavonoids, phenols, tannins and terpenoids are the major contributor to their α-amylase inhibitory potentials [38,40]. Several phytochemical constituents viz. alkaloid, terpenoids, flavonoids, phenols, saponins, tannins have been detected in *W. fruticosa* leaves [12,115]. Therefore, the inhibiting effect of *W. fruticosa* leaves on α-amylase may be largely attributable to the presence of these phytoconstituents. However, considering polyphenolic amounts and α-amylase inhibitory potentials, there was a positive association was observed in terms of TFC and amylase inhibitory activity. Crude extracts and fractions possessing higher flavonoid constitutes were seen to be more potent in inhibiting enzyme action. Flavonoids are a collection of hydroxylated phenolic phytocompounds that are proven to be potent free radical scavengers, and they have attracted great interest as possible therapeutics in treating free radical-mediated diseases, especially diabetes mellitus. They are said to influence carbohydrate digestion, insulin signaling, insulin secretion, and uptake of glucose in insulin-sensitive tissues by multifarious intracellular signaling pathways [116]. A considerable number of isolated flavonoids, i.e., luteolin, quercetin, quercetagetin, myricetin, hesperetin, etc., manifested promising α-amylase inhibitory potentials in several in vitro models [117,118,119]. Nair et al. (1976) and Kadota et al. (1990) were reported to isolate diverse flavonoid constituents including quercetin glycosides and myricetin glycosides from *W. fruticosa* leaves [70,120]. These flavonoids may have contributed significantly to the strong α-amylase inhibitory properties of NHFMEWF.

FST and TST are the two most common models of animals used in the screening of antidepressants as they are highly reliable and predictably validated [121]. Both of these tests are driven by the fact that when animals are exposed to short-term, unavoidable stress through suspension by the tail or being dropped into water, they develop an immobile behavior [122]. The animal immobility seen in these tests is called behavioral despair, and it is thought to reflect a human depression-like state that could be minimized by antidepressant agents [121,123]. These two tests are often very sensitive and quite specific to major antidepressants, including tricyclics, serotonin reuptake inhibitors, monoamine oxidase (MAO) inhibitors, and atypical antidepressants [123,124]. Additionally, in both models, the pharmacological antidepressant efficacy is also significantly interrelated with clinical potential for the tested drug [125]. Therefore, we preferred FST and TST to test the possible antidepressant effects of NHFMEWF and EAFMEWF. In our study, mice treated with the vehicle (control group) expressed passive actions (immobility behaviors) due to the stressful environment of the FST and TST, while mice treated with NHFMEWF or EAFMEWF at a dose of 100 mg/kg or 200 mg/kg reflected active actions (swimming and struggling behaviors) in both models by minimizing the immobility behaviors in a dose-dependent manner. In both models, a notable antidepressant-like effect was observed in EAFMEWF at a dosage of 200 mg/kg, which significantly shortened the duration of the depression-like state (immobile behavior) by over 50% (vs. control group) and is comparable to the positive control, fluoxetine-treated group (positive control). Fluoxetine, a routinely prescribed selective serotonin reuptake inhibitor (SSRI), reliably leads to the reduction in immobility behavior in both FST and TST [126,127]. In addition, FST, as a pharmacologically selective antidepressant treatment, induces two distinct active behavior patterns: SSRIs enhance the behavior of swimming while antidepressants act mainly to boost extracellular levels of norepinephrine/dopamine to maximize the behavior of climbing, suggesting that the swimming activity of the FST is mediated through serotonergic neurotransmission [128]. However, in TST, norepinephrine drugs and mixed serotonin-noradrenaline reuptake blockers are more effective than SSRIs, indicating possibly different neurochemical mechanisms mediating behavioral performance in these models [129]. GA, a polyphenol reported to be contained in *W. fruticosa* leaves, demonstrated antidepressant-like effects in a mice model via modulating the function of the serotonergic and catecholaminergic system, thus raising the levels of 5-HT and catecholamine in the synaptic clefts [130,131]. EA has also been demonstrated to possess antidepressant-like effects in FST and TST, which is reported to be mediated by adrenergic and serotonergic systems [132,133].

OS is likely to sharply engage in the pathogenesis of MDD through primary or secondary function [134]. This claim is reinforced by the fact that existing antidepressants may exert clinical effects other than monoamine level modulations. Indeed, several antidepressants are proven to minimize OS in both chronic-stress animal models and human studies [135]. There is evidence in humans and animals that antidepressant therapies suppress lipid peroxidation and normalizes dropped antioxidant levels [136]. Herken, et al. [137] stated that SSRIs (fluoxetine, citalopram, sertraline, and fluvoxamine) improved antioxidant system parameters, i.e., superoxide dismutase and adenosine deaminase levels, while NO and xanthine oxidase concentrations decreased in subjects with depression after 8 weeks of antidepressant treatment. Based on these studies, it is rational to anticipate the beneficial impact of antidepressants on antioxidant defenses, as multiple antioxidant mechanisms tend to reverse the potentially deleterious effects of ROS [138,139]. In this context, chemicals capable of modulating oxidative mechanisms possess the potential to prevent or treat MDD [55]. Polyphenols have shown antidepressant-like activity in multiple animal models of depression. Among multiple possible mechanisms, antioxidative actions of these molecules have been investigated to understand their antidepressant potential [140]. In this study and literature review, it was shown that the Dhaiphul possesses more polyphenolic compounds such as Woodfordin A-E, curcumin, resveratrol, fisetin, ferulic acid, and that quercetin are enriched in antioxidant capacity and have also been depicted to exert antidepressant-like activities [141]. Flavonoids also provoke antidepressant-like actions via antioxidant defenses against lipid peroxidation, leading to elevated 5-HT and norepinephrine levels in the central nervous system (CNS) [142]. Chhillar, et al. [143] publicized the antidepressant activity of GA, a potent antioxidant in unstressed and stressed mice, and proposed that the action could be mediated by mitigation in oxidative-nitrosative stress and inhibition of MAO-A. EA exerted antidepressant-like properties in stressed mice, potentially by inhibiting inducible NOS, thereby attenuating NO concentrations [133]. Based on this evidence, we speculate that EAFMEWF and NHFMEWF may modulate the monoaminergic system and suppress OS through ameliorating antioxidant mechanisms due to the presence of secondary metabolites like polyphenols and flavonoids, which may be a possible reason for this antidepressant activity.

Molecular docking is a well-established structural drug design technique that has been ubiquitously used to model interactions between drug molecules and target proteins at an atomic scale, and thereby allows us to outline the properties of the molecule at the active site of the target protein and to interpret the initial biochemical mechanisms [144]. This computational strategy is frequently considered for the design of molecules that address a diverse range of complicated pathologies [145]. The main focus of docking is to precisely predict the orientation of the ligand within the binding pocket of the target protein and to approximate the binding strength from the docking score. It allows us to contextualize the mechanism of action of nature-originated molecules as well as to rationalize their structure–activity relationships [146]. From this perspective, in the current study, we employed an in silico molecular docking method, with the intention of outlining the underlying possible biochemical mechanisms connected in the demonstrated antidiabetic (α-amylase inhibitory) and antidepressant-like activities of MEWF, NHFMEWF, and EAFMEWF. To validate our in vitro findings at the molecular level, six isolated compounds from *W. fruticosa* leaves were docked against the HPA and their binding energetics were investigated. By analyzing the docking score, we have found that compounds belonging to the subclass of flavonol glycosides and flavonol glycoside gallates possess a better affinity towards the active site of HPA. The docking scores of these compounds ranged from −7.41 to −6.105 kcal/mol which is comparable to the acarbose (docking score −7.752 kcal/mol). Glu233, Asp300, and His305 have been highlighted as the leading active site residues of HPA to interact with ligands. A previous study of the structure–activity relationship of flavonoids elucidated that flavonoids, particularly flavonols and flavones, which share a 4-oxo-flavonoid nucleus as the C-ring, are the most active inhibitors of α-amylase. They form a strongly conjugated π-system that boosts the stability of the compound as it binds to the active site of the α-amylase [117]. In sum, the potential α-amylase inhibitory action of NHFMEWF could be attributed to its high flavonoid content, which leads to an excellent binding affinity to the target enzyme.

The docking experiment, aimed at unraveling the underlying potential neurobiochemical mechanism involved in the antidepressant-like activity of *W. fruticosa*, focused on four target proteins: SERT3, DAT, MAO-A, and nNOS. Chemical neurotransmission is prompted through the Ca^2+^-mediated influx of neurotransmitters into the synaptic cleft (SC). Upon discharge into the SC, neurotransmitters such as 5-HT, dopamine, noradrenaline, glutamate, and GABA stimulate ligand-gated ion channels and G-protein-coupled receptors, leading to excitatory or inhibitory postsynaptic signaling cascades and currents [90]. SERT drops the levels of 5-HT at the SC by transferring them to the presynaptic cells, where they are either repackaged into secretory vesicles or metabolized by MAO-A [147]. An up-regulation of DAT, which contributes to more rapid reuptake of dopamine into the pre-synaptic neurons, was reported in patients having depression [148]. These transporters (SERT and DAT), including norepinephrine transporter, are top targets for many antidepressants. Interestingly, nevertheless, drugs aimed at a single transporter do not seem to be as clinically successful as those that block multiple transporters [149]. The current docking experiment concentrated on SERT3 and DAT has exposed that Q3DG, M3LA, Q3LA, and EA have substantial binding energies against both target transporters (SERT3 and DAT), suggesting their potential in the design of antidepressant therapies. Monoamine oxidases are mitochondrial-bound enzymes that catalyze the oxidative deamination of a variety of monoamines and play a prominent part in the metabolism of released neurotransmitters [150]. Any alteration (up-regulation or downregulation) in MAO levels may have catastrophic effects on the brain and behavior by dropping or elevating the levels of neurotransmitters and generating hazardous ROS [151]. These enzymes exist in two forms: type A and B monoamine oxidase (MAO-A and MAO-B). MAO-A catabolizes monoamine neurotransmitters (5-HT, dopamine, and norepinephrine), and serves as a central function in the onset, advancement, and therapeutic interventions of depressive disorders [152]. In light of this, we incorporated MAO-A into our docking analysis as a notable therapeutic target for depression, and the outcome disclosed that EA and GA possess greater potential for binding and interacting with active residues of MAO-A than the reference standard antidepressant (phenelzine). The impact of gallic acid on the MAO-A activity was previously investigated in animal models. Treatment (via i.p.) with GA significantly lowered MAO-A activity in mice [153], while the stress-induced spike in MAO-A activity was also significantly inhibited [143], which is consistent with our observations in docking analysis. NO is an unconventional gaseous neurotransmitter that controls and governs multiple vital physiological processes in the CNS, including mechanisms that may be linked to the pathogenesis of mental illnesses [154]. It is generated on demand by the NOS enzymes when L-arginine is converted to citrulline. It exists in three isoforms: endothelial NOS (eNOS), inducible NOS (iNOS), and neuronal NOS (nNOS) [154,155]. nNOS is prevalent in several brain regions involved with stress and depression, i.e., the hippocampus, hypothalamus, locus coeruleus, and dorsal raphe nucleus [156]. The rise in NO from nNOS exerted a negative impact on hippocampal neurogenesis and tends to be at least partly liable for the depressive-like behavior in animals [147]. Evidence indicates that reducing NO synthesis/levels by blocking NOS enzymes in the brain can exert antidepressant-like effects [156]. In this context, we docked the enrolled compounds against the nNOS enzyme, a potential target for antidepressant therapy. Analysis of the docking findings showed varied binding interactions between the compounds and the active site of nNOS, with docking scores varying from −3.699 to −8.449 kcal/mol. Q3DG demonstrated a maximum number of binding interactions with active site residues of nNOS with a docking score of −8.449 kcal/mol, followed by M3LA, Q3LA, EA, etc. Inhibition of inducible NOS activity by EA has previously been substantiated in animal models of depression [133]. Cumulative evidence and our docking analysis suggest that these enrolled polyphenols, including GA and EA, are at least partially involved in the demonstrated antidepressant-like activity of *W. fruticosa* leaves.

Over the past decades, merely a few drugs from hundreds of candidates eventually emerged in the market owing to the excessive failure rates at clinical trials [148]. The key factors that restricted 50% of drugs from entering the market were attributable to pharmacokinetic complications and toxicity in humans [149]. Therefore, it has now become evident that, in addition to the pharmacological characteristics, the ADME/Tox features are critical determinants of the optimum clinical efficacy of a drug [150]. Considering this perspective, we further researched the enlisted compounds for their ADME/Tox properties via SwissADME, pkCSM, and GUSAR Software. The physicochemical properties of compounds QA, MTMG, and EA were assessed to satisfy Lipinski’s RO5; however, flavonol glycosides and flavonol glycoside gallates compounds, i.e., Q3LA, M3LA, and Q3DG, violated RO5 (with violations ranging from 2 to 3). The RO5 is correlated with efficient aqueous solubility and intestinal permeability, and, when a compound fails to comply with the rules, there might be a high possibility that oral bioavailability problems will arise [151]. However, a significant class of drug molecules retrieved from natural products has been recorded to be RO5 outliers [157]. They are considered to be pharmacologically active and have desirable ADME/Tox properties, including decent oral bioavailability, even though they seldom meet the recommended “drug-likeness” parameters [158,159]. In addition to pharmacokinetic profiles, all compounds satisfied most of the absorption (excluding GI absorption) and metabolism parameters. They were also evaluated as non-substrates of P-gp., P-gp tends to have a greater effect on preventing the cellular absorption of drugs from the intestinal lumen to epithelial cells [160]. Therefore, the oral availability of the compounds tested would not be impaired by P-gp. In drug metabolism, the function of CYP1A2, CYP2C19, CYP2C9, CYP2D6, and CYP3A4 enzymes is remarkable, as they metabolize approximately 90% of drugs. Inhibition of CYP enzyme is the key mechanism for metabolic drug–drug interaction [156]. In this study, metabolism profiles of the compounds have demonstrated that the compounds are non-inhibitors of most CYP enzymes. Besides, the compounds had all toxicological properties, including Ames toxicity, hepatotoxicity, skin sensitization, and rat acute oral toxicity values within the ideal range. This study deduces that the enlisted polyphenolic compounds from *W. fruticosa* leaves possess desirable ADMET/Tox profiles. Taken together, the current data suggest promising depressive and hyperglycemic therapy protocols for NHFMEWF and EAFMEWF.

## 5. Conclusions

The current study provides evidence that *W. fruticosa* leaves possess dose-dependent antidepressant-like effects in predictive antidepressant models. At the higher dose, both fractions were shown to exert a significant effect on depressive-like behaviors of mice, and this effect may be driven by stimulation of the monoaminergic system and suppression of oxidative stress signaling pathways through the action of their bioactive constituents, in particular polyphenols. In the antidiabetic screening assay, the extract and fractions of *W. fruticosa* leaves inhibit the activity of α-amylase to varying degrees, which may be a potential underlying mechanism of its antihyperglycemic effect. Interestingly, the molecular docking analysis demonstrated binding affinities of previously isolated polyphenolic compounds from *W*. *fruticosa* leaves against five key therapeutic target proteins of DM2 and MDD, suggesting that flavonoid compounds may be a promising candidate in the development of anti-α-amylase and antidepressant therapeutics. However, further extensive research is required for the purification and modification of bioactive compounds from the leaves of *W. fruticosa* and to clarify their underlying molecular mechanisms.

## Figures and Tables

**Table 1:** Quantitative compounds retrieved from literature review of *W. fruticosa.*

Sl. No.	Compound Name	Plant Parts	Molecular Formula	Molecular Weight (g/mol)	Compound CID	Compound Class	References
1	Woodfordin A	Flowers	C_75_H_56_O_48_	1725.2	16130308	Tannin	[6,71,72]
2	Woodfordin I	Leaves	C_75_H_52_O_49_	1737.2	16130412	Tannin	[5,6]
3	Woodfordin B	Flowers	C_75_H_54_O_48_	1723.2	16130309	Tannin	[6,71,72]
4	Woodfordin C	Flowers	C_75_H_52_O_48_	1721.2	16131173	Tannin	[6,71,72]
5	Woodfordin D	Flowers	C_109_H_76_O_70_	2505.7	16131182	Tannin	[6,73]
6	Octacosanol	Stems	C_28_H_58_O	410.8	68406	Fatty alcohol	[4,6,74]
7	β-sitosterol	Stems	C29H50O	414.7	222284	Phytosterols	[4,6,74,75]
8	Hecogenin	Flowers	C_27_H_42_O_4_	430.6	91453	Triterpenoid	[4,6,75,76]
9	Meso-inositol	Flowers	C_6_H_12_O_6_	180.16	892	Phytosterols	[4,6,75,76]
10	Lupeol	Leaves	C_30_H_50_O	426.7	259846	Triterpenoid	[6,75,76]
11	Betulin	Leaves	C_30_H_50_O_2_	442.7	72326	Triterpenoid	[6,75,76]
12	Betulinic acid	Leaves	C_30_H_48_O_3_	456.7	64971	Triterpenoid	[6,75,76]
13	Oleanolic acid	Leaves	C_30_H_48_O_3_	456.7	10494	Triterpenoid	[6,75,76]
14	Ursolic acid	Leaves	C_30_H_48_O_3_	456.7	64945	Triterpenoid	[6,76]
15	Gallic acid	Leaves, flowers and stems	C_7_H_6_O_5_	170.12	370	Phenolic acid	[6,73,77,78,79]
16	Ellagic acid	Leaves and flowers	C_14_H_6_O_8_	302.19	5281855	Phenolic acid	[6,79,80]
17	Bergenin	Stems	C_14_H_16_O_9_	328.27	66065	Glycoside	[6,77]
18	Norbergenin	Stems	C_13_H_14_O_9_	314.24	73192	Glycoside	[6,77]
19	Chrysophanol-8-*O*-β-D glucopyranoside	Flowers	C_21_H_20_O_9_	416.4	442731	Glycoside	[4,6]
20	Lawsone	Leaves	C_10_H_6_O_3_	174.15	6755	Naphthoquinone	[6,81]
21	Quercetin 3-rhamnoside	Flowers	C_21_H_20_O_11_	448.4	5280459	Glycoside	[4,6]
22	Quercetin 3-β-L-arabinoside	Flowers and leaves	C_20_H_18_O_11_	434.3	10252339	Glycoside	[6,80]
23	Quercetin 3-*O*-β-L-arabinopyranoside	Leaves	C_26_H_28_O_15_	580.5	21722036	Glycoside	[6,73]
24	Quercetin 3-*O*-β-D-xylopyranoside	Leaves	C_20_H_18_O_11_	434.3	5320861	Glycoside	[6,73]
25	Quercetin 3-*O*-(6”-galloyl)- β-D- galactopyranoside	Leaves	C_28_H_24_O_16_	616.5	5491814	Glycoside	[6,73]
26	Quercetin 3-*O*-β-D-galactoside	Flowers and leaves	C_21_H_20_O_12_	464.4	5281643	Glycoside	[6,73]
27	Myricetin 3-*O*-α-L-arabinopyranoside	Leaves	C_37_H_58_O_10_	662.8	24721386	Glycoside	[6,73]
28	Quercetin 3-*O*-(6”-galloyl)- β-D- galactopyranoside	Leaves	C_28_H_24_O_16_	616.5	5491814	Glycoside	[6,77,79]
29	Naringenin 7-*O*-glucoside	Flowers	C_21_H_22_O_10_	434.4	92794	Glycoside	[4,6,79]
30	Kaempferol 3-*O*-glucoside	Flowers	C_21_H_20_O_11_	448.4	5282102	Myricetin glycosides, trihydroxyflavone	[4,6,79]
31	Pelargonidin 3,5-diglucoside	Flowers	C_27_H_31_ClO_15_	631	167642	Anthocyanidin pigment	[6,80]
32	Cyanidin 3,5-diglucoside	Flowers	C_27_H_31_O_16_	611.5	441688	Anthocyanidin pigment	[6,82]
33	1,2,3,6-tetra-*O*-galloyl- β-D-glucose	Flowers	C_34_H_28_O_22_	788.6	5153644	Tannin	[6,71,72,83,84,85]
34	1,2,4,6-tetra-*O*-galloyl-β-D-glucose	Flowers	C_34_H_28_O_22_	788.6	14464350	Tannin	[6,71,72,83,84,85]
35	1,2,3,4,6-penta-*O*-galloyl-β-D-glucose	Flowers	C_41_H_32_O_26_	940.7	65238	Tannin	[6,71,72,83,84,85]
36	Tellimagrandin I	Flowers	C_34_H_26_O_22_	786.6	442690	Tannin	[6,71,72,83,84,85]
37	Gemin D	Flowers	C_27_H_22_O_18_	634.5	471119	Tannin	[6,71,72,83,84,85]
38	Heterophylliin A	Flowers	C_34_H_26_O_22_	786.6	471120	Tannin	[6,71,72,83,84,85]
39	Oenothein B	Flowers	C_68_H_50_O_44_	1571.1	16132398	Tannin	[6,71,72,83,84,85]
40	Isoschimawalin A	Flowers	C_55_H_34_O_35_	1254.8	16130370	Tannin	[6,85]
41	Woodfordin E	Flowers	C_75_H_56_O_48_	1725.2	16130308	Tannin	[6,85]
42	Woodfordin F	Flowers	−	−	−	Tannin	[6,73,85]
43	Woodfordin G	Flowers	−	−	−	Tannin	[6,73,85]
44	Woodfordin H	Flowers	−	−	−	Tannin	[6,73,85]
45	Woodfruticosin	Leaves	C_75_H_52_O_48_	1721.2	16131173	Tannin	[6,71,72]
46	Oenothein-C	Flowers	C_34_H_24_O_22_	784.5	9962370	Flavanone	[86]
47	Naringenin	Flowers	C_15_H_12_O_5_	272.25	932	Flavanone	[79]
48	Kaempferol	Flowers	C_15_H_10_O_6_	286.24	5280863	Flavonoid	[79]
49	Quercetin	Flowers	C_15_H_10_O_7_	302.23	5280343	Flavonoid	[79]

**Table 2:** Nomenclature, abbreviation, PubChem CID, and molecular formula of the selected isolated phenolic compounds from *W. fruticosa* leaves for in silico studies.

Compound(s)	Abbreviation	PubChem CID	Molecular Formula
Gallic acid	GA	370	C_7_H_6_O_5_
Methyl tri-O-methylgallate	MTMG	15956	C_11_H_14_O_5_
Ellagic acid	EA	5281855	C_14_H_6_O_8_
Quercetin 3-O-α-L-arabinopyranoside	Q3LA	5481224	C_20_H_18_O_11_
Myricetin 3-O-α-L-arabinopyranoside	M3LA	44259439	C_20_H_18_O_12_
Quercetin 3-O-(6″-galloyl)-β-D-galactopyranoside	Q3DG	5491814	C_28_H_24_O_16_

**Table 3:** Total phenolic contents total flavonoid contents and DPPH free radical scavenging activities of methanol extract of *W. fruticosa* leaves and its derived fractions.

Sample(s)	Total Phenolic Content (mg GAE/gm of Dried Extract)	Total Flavonoid Content (mg QE/gm of Dried Extract)	IC_50_ (µg/mL)
MEWF	254.42 ± 1.53 *^a^*	41.44 ± 0.99 *^c^*	1.86 ± 0.16 *^a^*
NHFMEWF	16.63 ± 1.99 *^c^*	371.10 ± 1.99 *^a^*	47.03 ± 0.57 *^c^*
EAFMEWF	238.04 ± 1.01 *^b^*	97.11 ± 0.67 *^b^*	4.30 ± 0.28 *^b^*
AA	-	-	0.22 ± 0.02 *^a^*

Values are expressed as mean ± SEM (*n* = 3); values marked with different superscript letters (*a*, *b*, *c*) within a column signify statistical differences at *p* < 0.01 (Tukey’s HSD test); MEWF, methanol extract of *W. fruticosa* leaves; NHFMEWF, n-hexane fraction of methanol extract of *W. fruticosa* leaves; EAFMEWF, ethyl acetate fraction of methanol extract of *W. fruticosa* leaves; AA, ascorbic Acid; GAE, gallic acid equivalent; QE, quercetin equivalent; -, not estimated.

**Table 4:** Docking scores of the selected phenolic compounds from *W. fruticosa* leaves and reference standard drugs with HPA (PDB ID: 3BAJ) for antidiabetic activity and SERT3 (PDB ID: 5I6X), DAT (PDB ID: 4M48), MAO-A (PDB ID: 2Z5Y), and nNOS (PDB ID: 4UH5) for the antidepressant activity.

Protein(s)	HPA	SERT3	DAT	MAO-A	nNOS
PDB ID(s)	3BAJ	5I6X	4M48	2Z5Y	4UH5
Compound(s)	Docking Scores (kcal/mol)				
GA	−5.136	−5.859	−4.838	−7.86	−4.688
MTMG	−3.429	−4.782	−6.015	−6.532	−3.699
EA	−5.08	−6.415	−7.658	−7.951	−5.329
Q3LA	−6.84	−8.398	−9.794	-	−5.378
M3LA	−6.105	−7.378	−10.796	-	−5.912
Q3DG	−7.41	−8.678	−10.62	-	−8.449
Standard	−7.752	−9.426	−7.159	−6.782	-

GA, gallic acid; MTMG, methyl tri-O-methylgallate; EA, ellagic acid; Q3LA, quercetin 3-O-α-L-arabinopyranoside; M3LA, myricetin 3-O-α-L-arabinopyranoside; Q3DG, quercetin 3-O-(6″-galloyl)-β-D-galactopyranoside; Standard: Acarbose (HPA), Fluoxetine (SERT3), Bupropion (DAT), Phenelzine (MAO-A).

**Table 5:** Physicochemical properties of the six isolated compounds from *W. fruticosa* leaves.

Compound(s)	Lipinski Rules					Lipinski’s Violation(s)
	MW	HBA	HBD	LogP	nRB	
Acceptable Range	<500	≤10	≤5	≤5	≤10	
GA	170.12	5	4	0.21	1	0
MTMG	226.23	5	0	1.78	5	0
EA	302.19	8	4	1	0	0
Q3LA	434.35	11	7	0	3	2
M3LA	450.35	12	8	−0.48	3	2
Q3DG	616.48	16	10	−0.11	7	3

MW molecular weight; HBA number of H-bond acceptor; HBD number of H-bond donors; Log P, lipophilicity; nRB number of rotatable bonds; GA, gallic acid; MTMG, methyl tri-O-methylgallate; EA, ellagic acid; Q3LA, quercetin 3-O-α-L-arabinopyranoside; M3LA, myricetin 3-O-α-L-arabinopyranoside; Q3DG, quercetin 3-O-(6″-galloyl)-β-D-galactopyranoside.

**Table 6:** Pharmacokinetic, i.e., absorption and metabolism profiles of the selected compounds from *W. fruticosa* leaves.

Compound(s)	Absorption		Metabolism				
	GI Absorption	P-gp Substrate	CYP1A2 Inhibitor	CYP2C19 Inhibitor	CYP2C9 Inhibitor	CYP2D6 Inhibitor	CYP3A4 Inhibitor
GA	High	No	No	No	No	No	Yes
MTMG	High	No	No	No	No	No	No
EA	High	No	Yes	No	No	No	No
Q3LA	Low	No	No	No	No	No	No
M3LA	Low	No	No	No	No	No	No
Q3DG	Low	No	No	No	No	No	No

GI, gastrointestinal; P-gp, p-glycoprotein; CYP, cytochrome P450 enzymes; GA, gallic acid; MTMG, methyl tri-O-methylgallate; EA, ellagic acid; Q3LA, quercetin 3-O-α-L-arabinopyranoside; M3LA, myricetin 3-O-α-L-arabinopyranoside; Q3DG, quercetin 3-O-(6″-galloyl)-β-D-galactopyranoside.

**Table 7:** Toxicological profiles of the selected compounds from *W. fruticosa* leaves.

Compound(s)	Toxicological Parameters				
	Ames Toxicity	Hepatoxicity	Skin Sensitization	Rat Oral Acute Toxicity	
				LD_50_ (mg/kg)	Toxicity Classification
GA	No	No	No	1606	Class 4 in AD
MTMG	Yes	No	No	2815	Class 5 in AD
EA	No	No	No	1712	Class 4 in AD
Q3LA	No	No	No	2745	Class 5 in AD
M3LA	No	No	No	2748	Class 5 in AD
Q3DG	No	No	No	3501	Class 5 in AD

In AD, compounds fall within the applicability domain of models; GA, gallic acid; MTMG, methyl tri-O-methylgallate; EA, ellagic acid; Q3LA, quercetin 3-O-α-L-arabinopyranoside; M3LA, myricetin 3-O-α-L-arabinopyranoside; Q3DG, quercetin 3-O-(6″-galloyl)-β-D-galactopyranoside.

## Data Availability

All data generated or analyzed are contained within the present article.

## Supplementary Materials

The following are available online at https://www.mdpi.com/2223-7747/10/2/287/s1, Table S1. Key binding interactions of the studied compounds and standard drugs with active residues of the target proteins (HPA, SERT3, DAT, MAO-A, and nNOS); Figure S1. 2D visualization of the interactions of Q3LA, M3LA, and Acarbose with active site residues of HPA (PDB ID: 3BAJ); Figure S2. 2D visualization of the interactions of Q3LA and Fluoxetine with active site residues of SERT3 (PDB ID: 5I6X); Figure S3. 2D visualization of the interactions of Q3DG, Q3LA, EA, and Bupropion with active site residues of DAT (PDB ID: 4M48); Figure S4. 2D visualization of the interactions of GA, MTMG, and Phenelzine with active site residues of MAO-A (PDB ID: 2Z5Y); Figure S5. 2D visualization of the interactions of M3LA, Q3LA, and EA with active site residues of nNOS (PDB ID: 4UH5).

## Abbreviations

MEWF	methanol extract of *W. fruticosa* leaves
NHFMEWF	n-hexane fraction of methanol extract of *W. fruticosa* leaves
EAFMEWF	ethyl acetate fraction of methanol extract of *W. fruticosa* leaves
ADME/Tox	absorption, distribution, metabolism, excretion, and toxicity
DM2	type 2 diabetes mellitus
PPHG	postprandial hyperglycemia
HPA	human pancreatic α-amylase
ROS	reactive oxygen species
OS	oxidative stress
NADPH	nicotinamide adenine dinucleotide phosphate
MDD	major depressive disorder; 5-HT, 5-hydroxytryptamine
NO	nitric oxide
NOS	nitric oxide synthase
DNA	deoxyribonucleic acid; RT, room temperature
OD	optical density; Rpm, rotation per minute; p.o., orally; b.w., bodyweight
SERT3	serotonin transporter 3
DAT	dopamine transporter
MAO-A	monoamine oxidase A
nNOS	neuronal nitric oxide synthase
OPLS	optimized potentials for liquid simulations
RO5	rule of Five
ANOVA	analysis of variance
SPSS	statistical package for social science
GABA	gamma-aminobutyric acid; i.p., intraperitoneal
GI	gastrointestinal
P-gp	p-glycoprotein
CYP	cytochrome P_450_ enzymes
