# Supplementary material for: Antioxidant-Rich Woodfordia fruticosa Leaf Extract Alleviates Depressive-Like Behaviors and Impede Hyperglycemia
Source: Plants (Basel). 2021 Feb 3;10(2):287. doi: 10.3390/plants10020287 (PMC7913287; doi:10.3390/plants10020287)
Supplement: Supplementary file 1 [file plants-10-00287-s001.pdf]

Table S1. Key binding interactions of the studied compounds and standard drugs with active residues of the target proteins (HPA, SERT3, DAT, MAO-A, and nNOS).

Compound(s)	Hydrogen Bond Interactions	Hydrophobic Interactions
	Interacting Residues (Distance, Å)	Interacting Residues (Type of bond)
**Human Pancreatic α-Amylase (PDB ID: 3BAJ)**		
Q3DG	Asp197 (3.98), Asp197 (4.11), Glu233 (4.48), Glu233 (4.94), His305 (3.13)	Trp59 (pi-pi stacked),His201 (pi-pi t-shaped), Leu162 (Two pi-alkyl), Ile235 (pi-alkyl)
Q3LA	Trp59 (4.18), Asp197 (3.77), Asp197 (4.2), His299 (5.58), Asp300 (4.49), His305 (4.02)	-
M3LA	Thr163 (3.52), Arg195 (7.22), Asp197 (4.00), Lys200 (4.88), Glu233 (4.64), His299 (5.10), Asp300 (4.07), His305 (3.49), His305 (4.16)	Tyr151 (pi-pi stacked), His201 (pi-pi t-shaped), Leu162 (pi-alkyl), Lys200 (pi-alkyl), Ile235 (pi-sigma)
GA	Glu233 (3.57), Glu233 (4.19)	His201 (pi-pi t-shaped), Leu162 (pi-alkyl), Lys200 (pi-alkyl), Ile 235 (pi-alkyl)
EA	Glu233 (3.92), Glu233 (4.08), His305 (2.95), His305 (3.52)	Tyr62 (pi-pi t-shaped), Leu162 (pi-alkyl), Ala198 (pi-alkyl)
MTMG	Tyr151 (6.35)	His201 (pi-pi t-shaped), Leu162 (alkyl), Lys200 (Two alkyl), Ile 235 (Two alkyl), Tyr151 (pi-alkyl), Leu162 (pi-alkyl), Ile 235 (pi-alkyl)
Acarbose	Trp59 (4.49), Thr163 (4.30), Arg195 (7.09), Lys200 (4.81), Glu233 (3.96), Glu240 (5.32), Asp300 (4.06), Asp300 (4.53), His305 (3.23), His305 (5.84)	Leu165 (Two alkyl), His101 (pi-alkyl)
**Serotonin Transporter 3 (PDB ID: 5I6X)**		
Q3DG	Asp98 (1.79), Asp98 (2.00), Asp98 (2.14), Thr497 (1.86)	Phe335 (Two pi-pi stacked), Phe556 (pi-pi stacked), Tyr95 (pi-pi t-shaped), Arg104 (pi-alkyl), Ala331 (pi-alkyl)
Q3LA	Asp98 (1.67), Phe335 (2.88)	Tyr176 (Two pi-pi t-shaped), Ala169 (pi-alkyl), Ile172 (Two pi-alkyl), Ala173 (pi-alkyl)
M3LA	Asp98 (1.66), Arg104 (2.60), Tyr175 (2.06), Phe335 (2.23), Glu494 (2.15)	Phe335 (pi-pi stacked), Ile172 (pi-alkyl), Val501 (pi-alkyl)
EA	Tyr175 (1.69), Gln332 (1.85), Thr497 (2.07)	Phe335 (Four pi-pi stacked), Arg104 (pi-alkyl), Ala331 (pi-alkyl)
GA	Tyr95 (1.84), Tyr95 (2.20), Ala96 (2.79), Ser438 (2.73)	Tyr95 (pi-pi stacked)
MTMG	-	Ile172 (alkyl), Val501 (alkyl), Tyr95 (pi-alkyl), Phe341 (pi-alkyl)
Fluoxetine	Tyr95 (1.75)	Tyr176 (pi-pi t-shaped), Phe441 (pi-pi t-shaped), Ile172 (alkyl), Ala173 (alkyl), Ile172 (Two pi-alkyl), Val501 (pi-alkyl)
**Dopamine Transporter (PDB ID: 4M48)**		
M3LA	Phe43 (1.90), Asp46 (1.63), Phe319 (1.70), Phe325 (2.03), Asp475 (2.11), Asp475 (2.48)	Phe325 (pi-pi stacked), Tyr123 (pi-pi t-shaped), Ala117 (pi-alkyl), Val120 (Two pi-alkyl), Ala479 (Two pi-alkyl)
Q3DG	Trp51 (2.97), Tyr123 (1.91), Phe325 (1.93), Asp475 (1.66), Asp475 (1.88), Asp475 (2.17), Asp475 (3.56), Arg476 (2.52)	Phe325 (Two pi-pi stacked), Ala117 (pi-alkyl), Val120 (Two pi-alkyl), Ala479 (Two pi-alkyl)
Q3LA	Phe43 (2.00), Phe325 (2.13), Asp475 (1.84), Asp475 (2.08)	Phe325 (pi-pi stacked), Tyr123 (pi-pi t-shaped), Ala117 (pi-alkyl), Val120 (Two pi-alkyl), Ala479 (Three pi-alkyl), Ile483 (pi-alkyl)
EA	Asp46 (1.77), Phe325 (2.38)	Phe325 (Three pi-pi stacked), Tyr124 (pi-pi t-shaped), Val120 (Three pi-alkyl), Ala479 (pi-alkyl)
MTMG	-	Phe325 (pi-pi stacked), Ile116 (alkyl), Ala117 (alkyl), Val120 (alkyl), Ala479 (alkyl), Ile483 (Two alkyl), Val120 (pi-alkyl), Tyr124 (Two pi-alkyl), Phe319 (pi-alkyl), Phe325 (two pi-alkyl)
GA	Asp46 (1.86), Asp46 (1.96) Phe319 (3.06)	Phe325 (pi-pi stacked)
Bupropion	Phe319 (2.96)	Phe325 (pi-pi stacked), Ala117 (alkyl), Val120 (alkyl), Val120 (pi-alkyl), Phe325 (pi-alkyl), Ala479 (pi-alkyl)
**Monoamine Oxidase A (PDB ID: 2Z5Y)**		
EA	Phe208 (2.72), HOH884 (2.40)	Tyr407 (Two pi-pi stacked), Ile180 (pi-alkyl), Ile335 (pi-alkyl)
GA	Ala68 (1.60), Tyr69 (1.73), HOH884 (1.69)	Tyr407 (pi-pi stacked), Tyr444 (pi-pi stacked)
MTMG	-	Tyr407 (pi-pi stacked), Tyr444 (pi-pi stacked), Lys305 (alkyl), Phe352 (pi-alkyl), Tyr407 (Three pi-alkyl),Tyr444 (pi-alkyl)
Phenelzine	Ile180 (2.04), Asn181 (1.92), Gln215 (2.75)	Ile180 (pi-alkyl), Ile335 (pi-alkyl), Leu337 (pi-alkyl)
**Neuronal Nitric Oxide Synthase (PDB ID: 4UH5)**		
Q3DG	His342 (2.75), Gln483 (5.86), Gly591 (3.00), Trp592 (2.82), Glu597 (2.22), Glu597 (2.55), Asp602 (4.46), Asp605 (1.66), Asp605 (1.89), Ser607 (2.89), Arg608 (1.97), Hem750 (1.90), Hem750 (2.78)	Hem750 (pi-pi stacked), Pro570 (pi-alkyl)
M3LA	Gln483 (2.27), Asn574 (1.70), Tyr593 (2.26), Trp593 (3.09), Glu597 (1.64), Glu597 (2.45), Arg601 (5.02), Asp602 (2.96), Asp605 (2.27), Hem750 (2.18)	-
Q3LA	Trp311 (2.04), Trp311 (2.11), Gln483 (2.69), Ser607 (2.72), Asp714 (1.58), Asp714 (1.61), Hem750 (2.21), Hem750 (2.57)	-
EA	Gln483 (2.38), Tyr593 (2.42), Asp602 (1.60), Asp602 (1.83), Hem750	Trp683 (pi-pi t-shaped)

	(1.68), Hem750 (2.26)	
GA	Tyr567 (1.67), Trp593 (2.71), Glu597 (1.82), Hem750 (1.86)	Val572 (pi-alkyl)
MTMG	Tyr567 (2.68)	Pro570 (alkyl), Val572 (Two alkyl), Val572 (pi-alkyl), Phe589 (pi-alkyl), Tyr593 (pi-alkyl), Hem750 (Two pi-alkyl)

GA, gallic acid; MTMG, methyl tri-O-methylgallate; EA, ellagic acid; Q3LA, quercetin 3-O-α-L-arabinopyranoside; M3LA, myricetin 3-O-α-L-arabinopyranoside; Q3DG, quercetin 3-O-(6''-galloyl)-β-D-galactopyranoside.

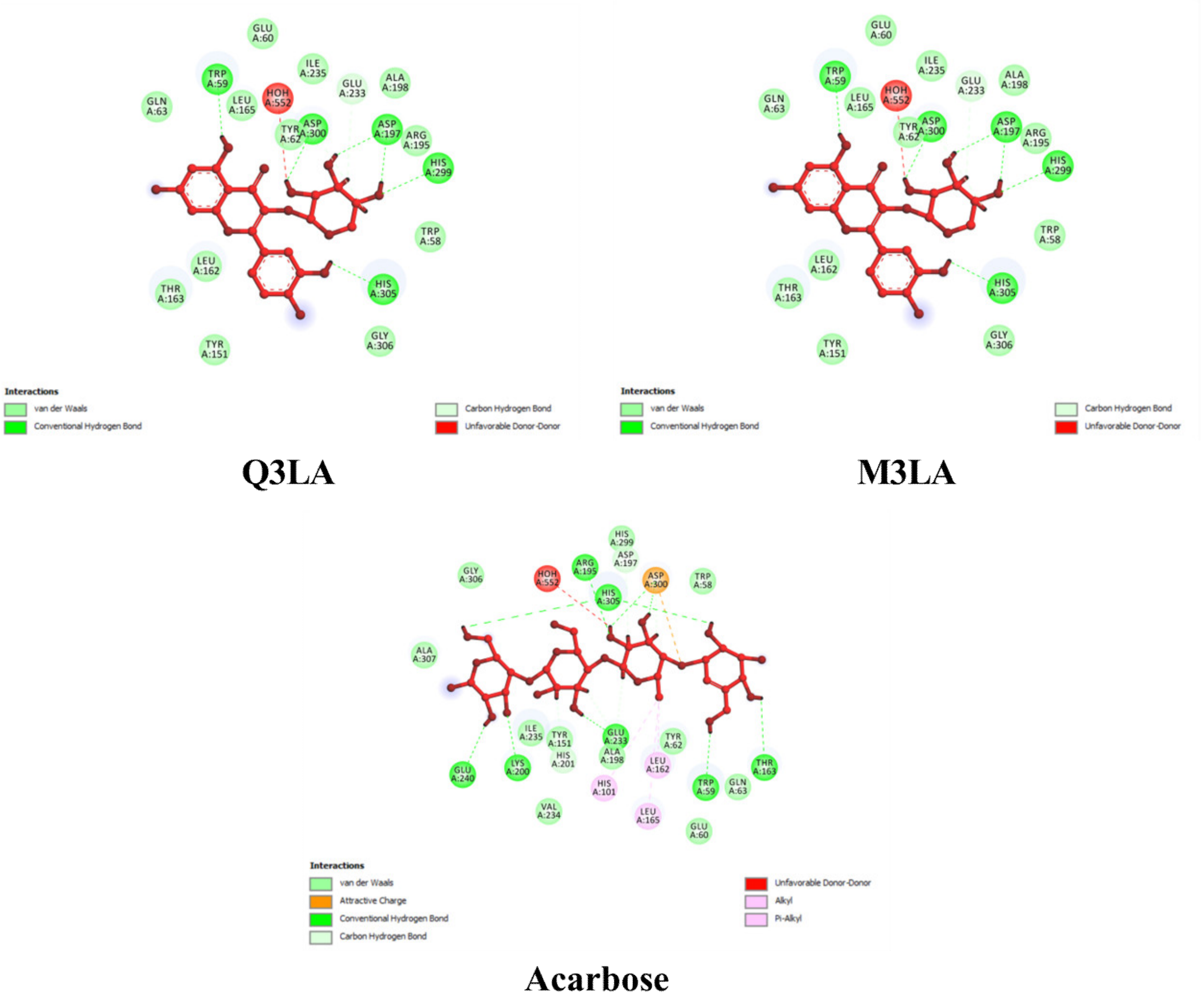

Figure S1. 2D visualization of the interactions of Q3LA, M3LA, and Acarbose with active site residues of HPA (PDB ID: 3BAJ). Q3LA, quercetin 3-O-α-L-arabinopyranoside; M3LA, myricetin 3-O-α-L-arabinopyranoside.

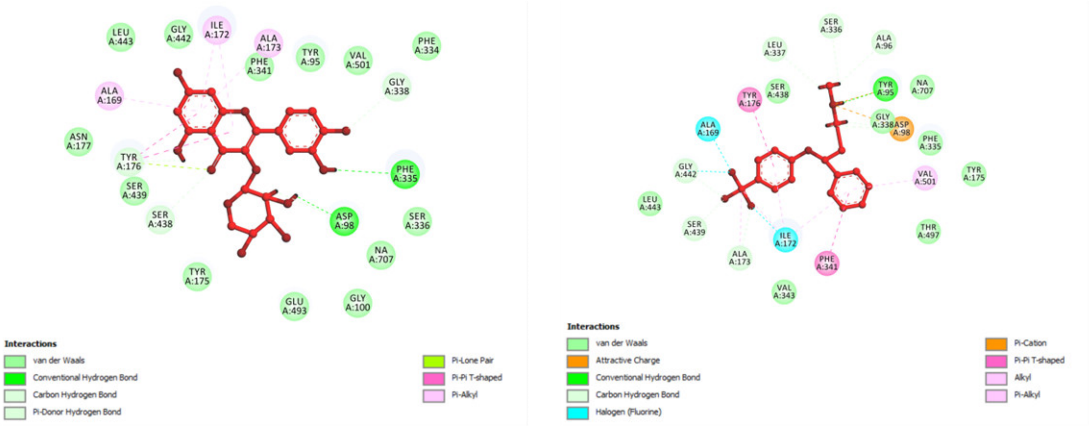

Q3LA **Fluoxetine**

Figure S2. 2D visualization of the interactions of Q3LA and Fluoxetine with active site residues of SERT3 (PDB ID: 5I6X). Q3LA, quercetin 3-O-α-L-arabinopyranoside.

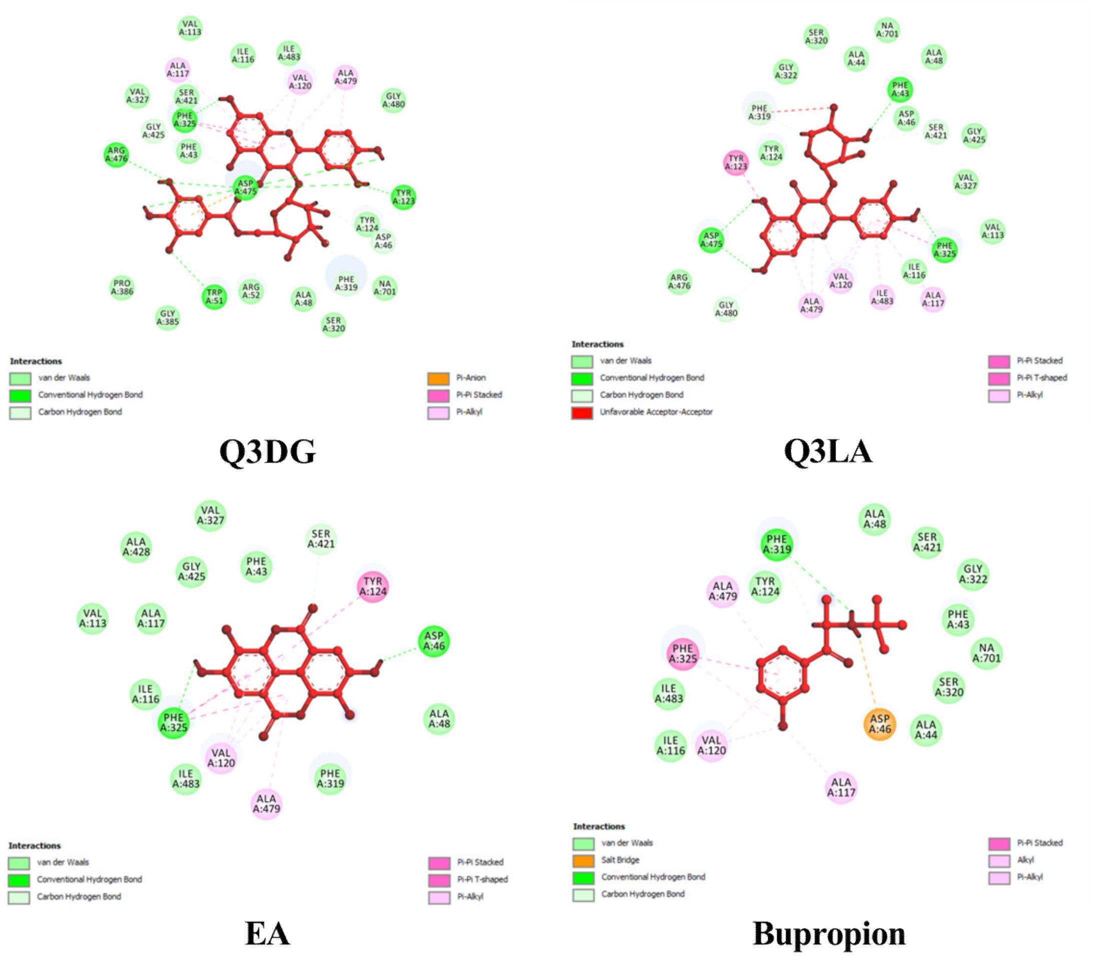

Figure S3. 2D visualization of the interactions of Q3DG, Q3LA, EA, and Bupropion with active site residues of DAT (PDB ID: 4M48). Q3DG, quercetin 3-O-(6"-galloyl)-β-D-galactopyranoside; Q3LA, quercetin 3-O-α-L-arabinopyranoside; EA, ellagic acid.

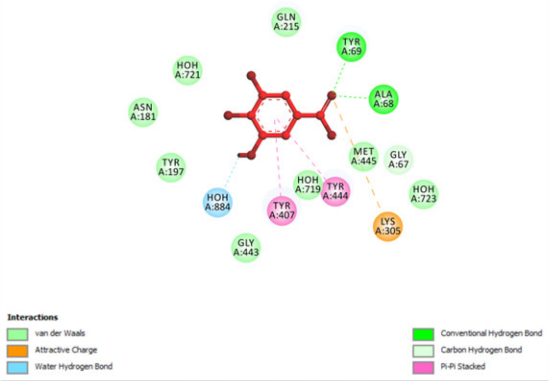

GA

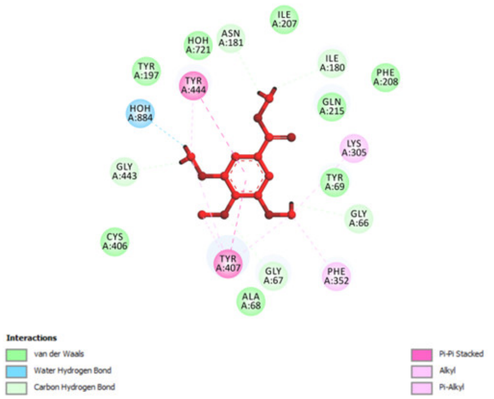

MTMG

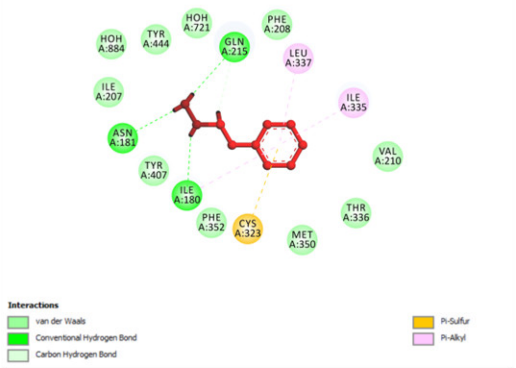

Phenelzine

Figure S4. 2D visualization of the interactions of GA, MTMG, and Phenelzine with active site residues of MAO-A (PDB ID: 2Z5Y). GA, gallic acid; MTMG, Methyl tri-O-methylgallate.

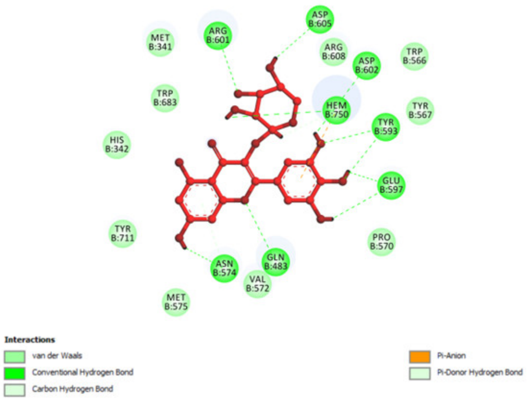

M3LA

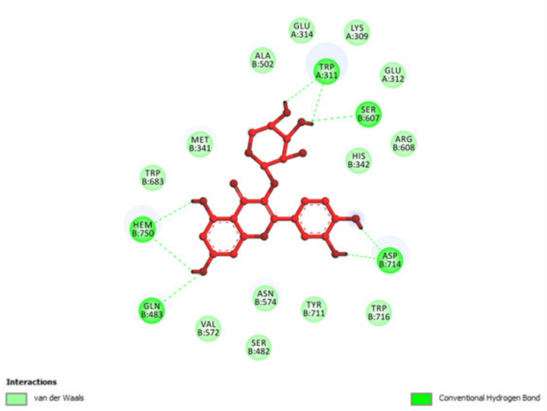

Q3LA

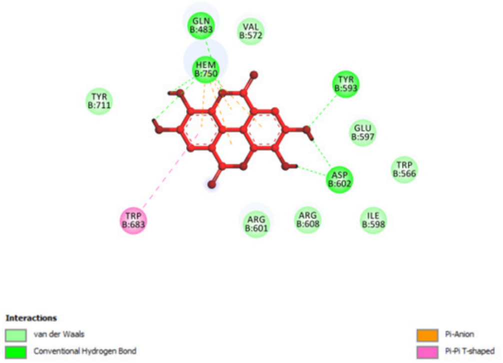

EA

Figure S5. 2D visualization of the interactions of M3LA, Q3LA, and EA with active site residues of nNOS (PDB ID: 4UH5). M3LA, myricetin 3-O-α-L-arabinopyranoside; Q3LA, quercetin 3-O-α-L-arabinopyranoside; EA, ellagic acid.
